# BRAT1 - a new therapeutic target for glioblastoma

**DOI:** 10.1007/s00018-024-05553-0

**Published:** 2025-01-21

**Authors:** Alicia Haydo, Jennifer Schmidt, Alisha Crider, Tim Kögler, Johanna Ertl, Stephanie Hehlgans, Marina E. Hoffmann, Rajeshwari Rathore, Ömer Güllülü, Yecheng Wang, Xiangke Zhang, Christel Herold-Mende, Francesco Pampaloni, Irmgard Tegeder, Ivan Dikic, Mingji Dai, Franz Rödel, Donat Kögel, Benedikt Linder

**Affiliations:** 1 Experimental Neurosurgery, Department of Neurosurgery, Neuroscience Center, Goethe University Hospital, Goethe University Frankfurt, 60528 Frankfurt am Main, Germany; 2 Department of Radiotherapy and Oncology, Goethe University Hospital, 60590 Frankfurt am Main, Germany; 3 Institute of Biochemistry II, Goethe University, Frankfurt am Main, Germany; 4 Department of Structural Biology, St. Jude Children’s Research Hospital, Memphis, USA; 5 Department of Chemistry, Purdue University, West Lafayette, IN 47907 USA; 6 Division of Experimental Neurosurgery, Department of Neurosurgery, University Hospital Heidelberg, 69120 Heidelberg, Germany; 7 Buchmann Institute for Molecular Life Sciences, Goethe University Frankfurt, 60438 Frankfurt am Main, Germany; 8 Institute for Clinical Pharmacology, Faculty of Medicine, Goethe University Frankfurt, Frankfurt am Main, Germany; 9 Cardio-Pulmonary Institute, 60590 Frankfurt am Main, Germany; 10 Department of Chemistry and Winship Cancer Institute, Emory University, Atlanta, GA 30022 USA; 11 Frankfurt Cancer Institute, Goethe University, 60590 Frankfurt am Main, Germany; 12 German Cancer Consortium (DKTK), Partner Site Frankfurt, 60590 Frankfurt am Main, Germany; 13 German Cancer Research Center DKFZ, 69120 Heidelberg, Germany; 14 Present Address: Radiation Biology and DNA Repair, Darmstadt, TU Germany

**Keywords:** Brain tumor, Tumor stemness, Curcusone D, Tumor invasion, Cell migration, DNA damage response

## Abstract

Glioblastoma (GBM), the most malignant primary brain tumor in adults, has poor prognosis irrespective of therapeutic advances due to its radio-resistance and infiltrative growth into brain tissue. The present study assessed functions and putative druggability of BRCA1-associated ATM activator 1 (BRAT1) as a crucial factor driving key aspects of GBM, including enhanced DNA damage response and tumor migration. By a stable depletion of BRAT1 in GBM and glioma stem-like (GSC) cell lines, we observed a delay in DNA double-strand break repair and increased sensitivity to radiation treatment, corroborated by in vitro and in vivo studies demonstrating impaired tumor growth and invasion. Proteomic and phosphoproteomic analyses further emphasize the role of BRAT1’s cell migration and invasion capacity, with a notable proportion of downregulated proteins associated with these processes. In line with the genetic manipulation, we found that treatment with the BRAT1 inhibitor Curcusone D (CurD) significantly reduced GSC migration and invasion in an ex vivo slice culture model, particularly when combined with irradiation, resulting in a synergistic inhibition of tumor growth and infiltration. Our results reveal that BRAT1 contributes to GBM growth and invasion and suggest that therapeutic inhibition of BRAT1 with CurD or similar compounds might constitute a novel approach for anti-GBM directed treatments.

**Supplementary Information:**

The online version contains supplementary material available at 10.1007/s00018-024-05553-0.

## Introduction

Tumors originating in the central nervous system pose significant challenges due to the delicate nature of the brain. The brain possesses unique physiological features such as the blood-brain barrier and specialized neuron and diverse glial cell types. Therefore, a phenotypically and genotypically distinction is made between WHO grade 2 and 3 diffuse or anaplastic astrocytomas or oligodendrogliomas, grade 4 diffuse pediatric gliomas and glioblastomas (GBM). These entities are further divided into different subgroups according to their genotypic characteristics [1–4]. Among those cancers, GBMs represent the most aggressive malignant tumor type, with a median survival rate of only 15 months despite maximally possible treatment [5, 6]. This is primarily caused by early relapses due to diffusely infiltrating tumor cells migrating as single cells or small clusters into the neuropil (network of intertwined neurons and glia cells). Therefore, patients cannot be completely cured by surgical resection of these tumors [6, 7]. GBMs are thus routinely treated with a combination of radiation and chemotherapy (RCT) [8], despite showing limited responses to this multimodal treatment [6].

Treatment failures are mostly caused by the infiltration of tumor cells into the surrounding brain tissue, preventing complete tumor resection and leading to inevitable recurrence, largely attributed to treatment-resistant glioma stem-like cells (GSCs) [9]. The invasive behaviour of GBM cells is facilitated by complex interactions between tumor cells and the surrounding microenvironment. Signaling pathways such as the mitogen-activated protein kinase (MAPK)/ extracellular signal-regulated kinase (ERK), phosphoinositide-3-kinase (PI3K)/ Akt/ mechanistic target of rapamycin (mTOR), and wingless/integrated (Wnt) play critical roles in regulating cytoskeletal dynamics, cell adhesion, and extracellular matrix remodelling, thereby promoting GBM cell migration and invasion [10]. Additionally, transcription factors such as TWIST1 and SNAIL1 promote epithelial-mesenchymal transition (EMT), a process associated with increased invasiveness and stemness in GBM and other cancer entities [11, 12]. Furthermore, GBM cells have been observed to migrate along existing brain structures, including white matter tracts, blood vessels, and the extracellular matrix, facilitated by interactions with multiple transmembrane receptors [13]. GSCs, distinguished by their increased differentiation potential compared to other tumor cells, typically reside within specialized niches and express various stemness-associated marker proteins. The abundance of GSCs, which possess enhanced migratory capacity and therapy resistance, further exacerbates the invasive behaviour of GBM [9, 14].

Further, it has been shown that GSCs are particularly resistant to radiotherapy due to alterations in the DNA damage response (DDR) pathway, which facilitate DNA damage checkpoint activation and allow the tumor cells to survive in a quiescent state [15, 16]. It was shown that the phenotypic heterogeneity arises from non-hierarchical, reversible state transitions, instructed by the microenvironment. Therefore, targeting this inherent cancer cell plasticity emerges as a novel relevant target for treatment [17]. For this, we have recently shown that inhibiting the Hedgehog and NOTCH pathways in GSCs with Arsenic Trioxide (ATO) in combination with the anticancer agent (-)-Gossypol (Gos), which reduces not only stemness markers/properties, but also decreases expression of proteins involved in DNA repair and cell migration. One protein, which was prominently depleted in this study was the BRCA1-associated ATM activator 1 (BRAT1) [18]. BRAT1 is ubiquitously expressed and interacts with BRCA1 and ATM and is therefore potentially involved in the DDR, cell proliferation, cell migration and invasion [19, 20]. Interestingly, BRAT1 variants are associated with diverse clinical conditions, from neonatal seizures to neurodevelopmental disorders [21], but it´s potential role in brain tumors is largely unknown. Recently, it was shown that the integrator subunit complexes INTS11 and INTS9 interact with BRAT1 and form a trimeric complex in human HEK293T cells as well as in the pluripotent human embryonal carcinoma cell line (NT2) to regulate the expression of neurodevelopmental genes [22]. A further study showed that the interaction of BRAT1 and the subunits of the Integrator complex (INTS9/INTS11) are responsible for processing the 3’ ends of various noncoding RNAs and pre-mRNAs [23].

The curcusone diterpenes, isolated from *Jatropha curcas*, share a tricyclic carbon skeleton with the daphnane and tigliane diterpenes and have shown promising anticancer properties and BRAT1 was identified as a key cellular target of the curcusones using chemoproteomics [24]. Therefore, exhibiting potential antitumor effects are probably mediated at least in part by interfering with BRAT1/integrator subunit complexes (INTS11/INTS8) [25] and thereby blocking promoter activity and likely transcription of BRAT1 itself. Curcusone D (CurD) leads to an impaired DDR, reduced cancer cell migration, and potentiated activity of the DNA damaging drug etoposide, in HeLa, MCF-7 and MDA-MB-231 cells, indicating a potential role of BRAT1 in therapy responses and tumor growth. Importantly, CurD was described to reduce the levels of BRAT1 through proteasomal degradation providing a pharmacologic tool to further assess the role of BRAT1 in GBM pathology [24]. This finding is significant as BRAT1 has been considered a non-pharmaceutical targetable oncoprotein with no known small molecule inhibitors.

Understanding the molecular mechanisms driving GBM invasion and migration is crucial for the development of novel therapeutic strategies. Moreover, advances in imaging techniques, like OTCxLSFM, provide valuable tools for studying GBM invasion dynamics and evaluating the efficacy of anti-invasive therapies [26]. Elucidating the complexities of GBM cell invasion and migration may lead to the development of more effective therapeutic interventions for this devastating disease. Here we show that BRAT1 plays a pivotal role in regulating cancer cell behaviour by impacting both migration and invasion, while also exerting an influence on the DDR. In particular, we identify BRAT1 as a novel critical factor contributing to the aggressive pro-migratory and pro-invasive growth characteristics of GBM in brain tissue. Lastly, we describe the BRAT1 inhibitor CurD as a putative drug candidate specifically targeting BRAT1, which partially increases radiosensitivity of GBM, thereby opening new perspectives for the development of targeted GBM therapy.

## Results

### BRAT1 expression is elevated in GBM

**Fig. 1:**
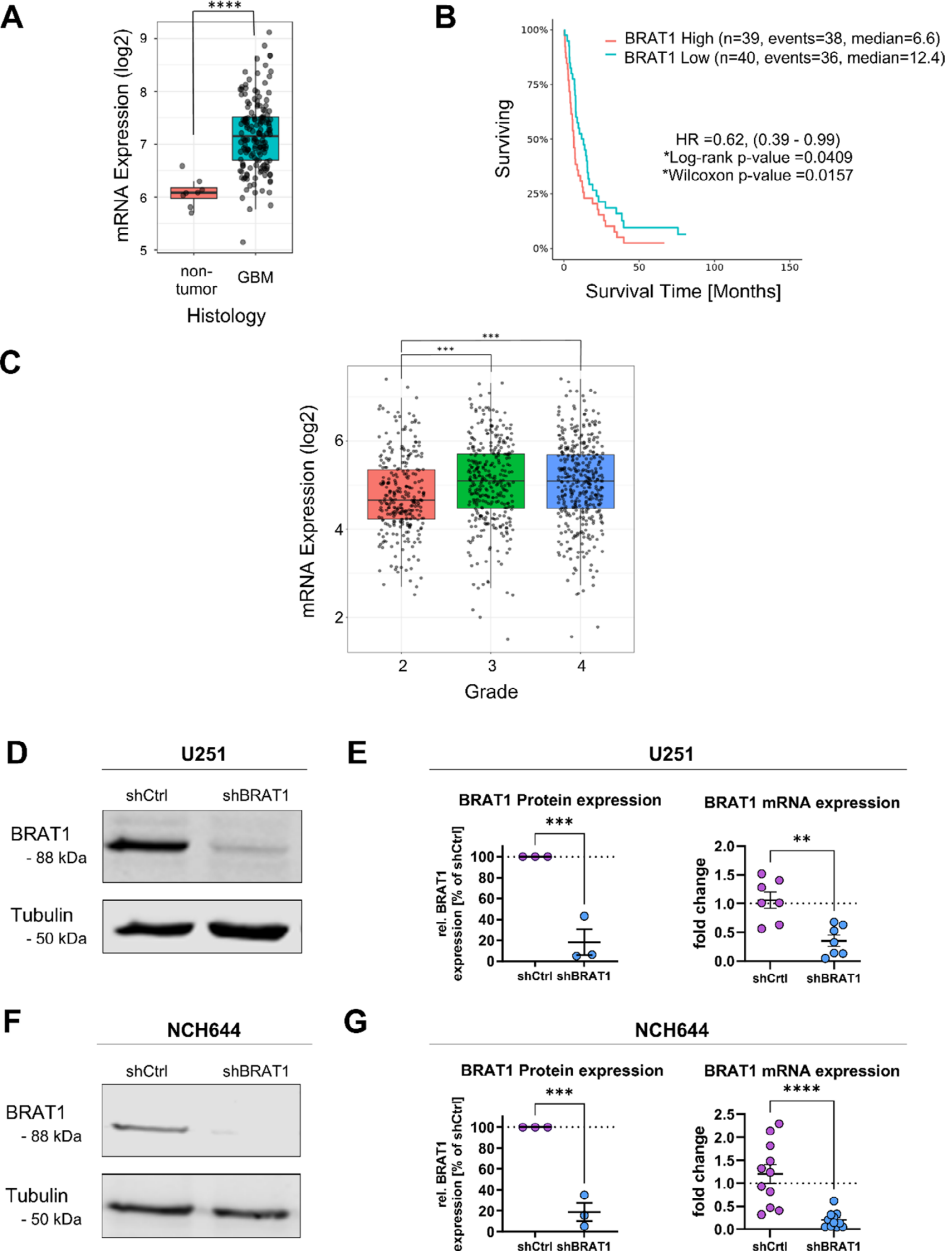
BRAT1 expression is increased in GBM. (**A**) Increased levels of BRAT1 compared to healthy tissue. Analysis of Gravendeel-dataset [27] using the GlioVis portal [28]. (**B**) Higher BRAT1 levels are associated with decreased GBM patient survival. (**C**) According to the CGGA dataset [29] BRAT1 expression is elevated in higher grade tumors. (**D**) Western blot of stable shRNA BRAT1 depletion in U251 GBM cells, Tubulin as housekeeping protein. (**E**) U251 BRAT1 densitometric protein validation (*n* = 3 biological replicates) and mRNA expression (*n* = 3 in minimum technical duplicates). (**F**) Western blot and densitometric validation of stable shRNA BRAT1 depletion in NCH644 GSCs; Tubulin as housekeeping protein. (**G**) NCH644 BRAT1 densitometric protein validation (*n* = 3 biological replicates) and mRNA expression (*n* = 3 in minimum technical triplicates). Original western blots used for quantification are available via zenodo.org (10.5281/zenodo.14390501) [32]. Statistics: A: Unpaired two-sided t-test between group levels; B: Survival analysis using Wilcoxon signed rank test. C: One-way ANOVA with posthoc t-test using a correction of alpha according to Dunnett versus control. E and G: Unpaired two-sided t-test between groups. Error bars are SEM. *: *p* < 0.05; **: *p* < 0.01; ***: *p* < 0.001; ****: *p* < 0.0001

Recently, we applied a combination of two anticancer drugs ATO and Gos, which showed that the combination leads to profound reduction of proteins involved in the DDR, as well as 40% of the proteins, which were downregulated, cluster under the term movement. In this dataset, one of the most prominent downregulated protein was BRAT1 [18]. An analysis of publicly available datasets revealed, that BRAT1 is overexpressed in GBM compared to healthy tissues (Fig. 1A) and it correlates negatively with patient survival according to the Gravendeel-dataset [27] analyzed via the GlioVis portal [28] (Fig. 1B). This shows, that BRAT1 is routinely expressed in GBM tumors, with an additional impact of comparatively higher BRAT1 expression for patient outcome. Further, an analysis of the more recent CGGA database [29] revealed that BRAT1 expression increases with tumor grade (Fig. 1C). These data suggest that BRAT1 is an as-of-yet underappreciated factor for GBM aggressiveness. To investigate the potential effects of BRAT1, stable BRAT1-knockdown (KD) cells were developed using lentiviral shRNA constructs for the GBM cell line U251 [30] and the GSC line NCH644 [31], which represents a sub-population inside of GBM tumors, by expressing various markers associated with stemness [9]. The U251 shBRAT1 cell line showed a clear reduction in BRAT1 protein expression (residual BRAT1 expression ~ 15% in shBRAT1 cells), confirmed by densitometric quantification, as well as a decreased mRNA expression of *BRAT1* compared to shCtrl cells, displaying a reduction of ~ 60% (shBRAT1 ~ 0.4-fold expression) compared to the control (Fig. 1D and E). To validate the KD efficiency in NCH644 BRAT1 depleted GSCs, we could demonstrate significant reduction in BRAT1 protein and mRNA expression as well. Specifically, protein expression was diminished to ~ 20% compared to the control, while mRNA expression showed a reduction of ~ 75% (shBRAT1 ~ 0.25-fold expression) compared to the control (Fig. 1F and G). Original western blots used for KD quantification are available via zenodo.org (10.5281/zenodo.14390501) [32]. By generating these KD cell lines, we aimed to investigate in the following experiments whether an altered DDR or migratory capacity could be observed in U251 cells and NCH644 cells following BRAT1 depletion.

### BRAT1 is essential for an efficient DNA repair

**Fig. 2:**
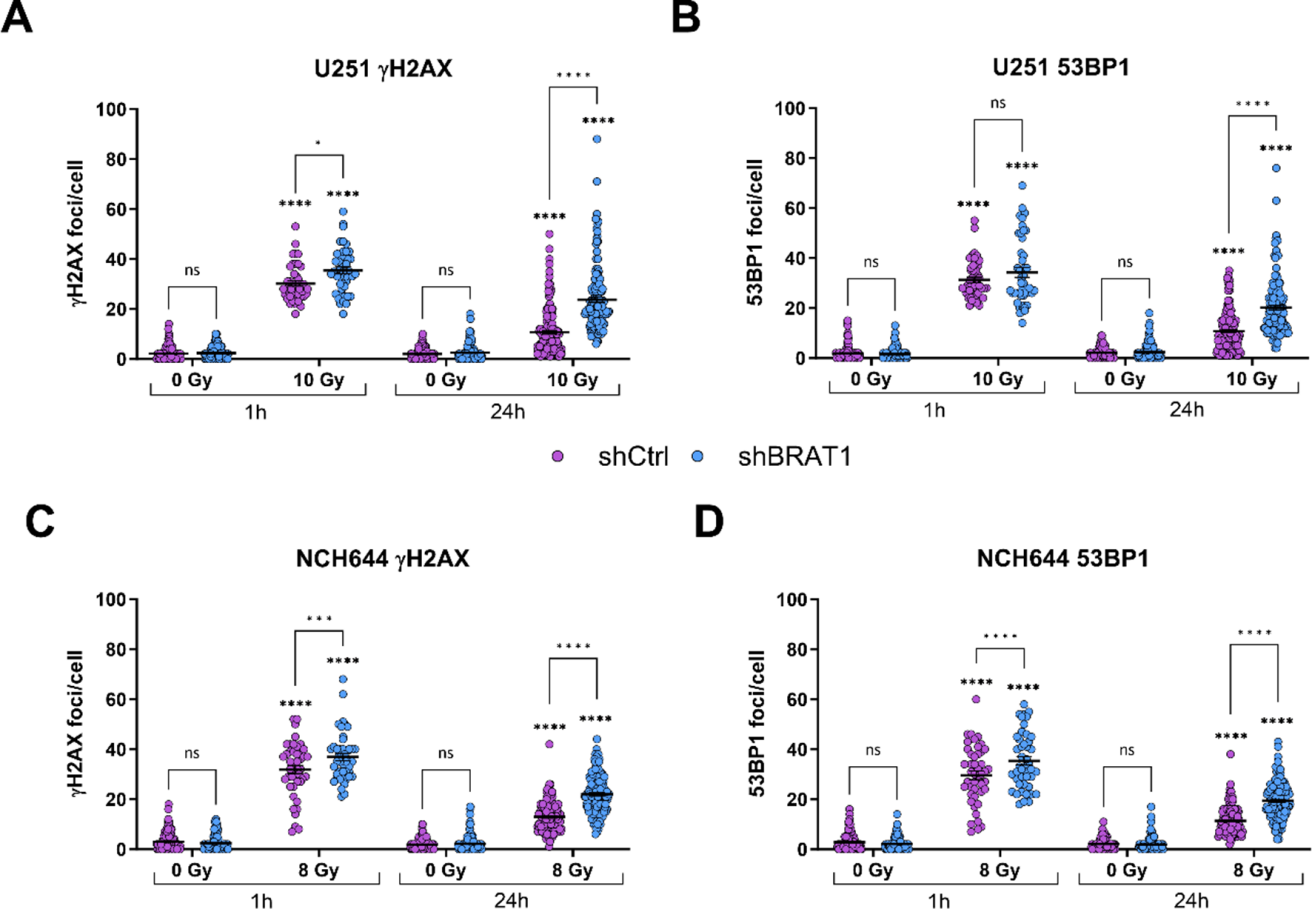
BRAT1 is essential for efficient DNA repair. shCtrl and shBRAT1 cells were compared in two cell lines. (**A**) U251 γH2AX foci assays and (**B**) U251 53BP1 foci assays 1 h and 24 h after 10 Gy IR. (**C**) NCH644 γH2AX foci assays and (**D**) NCH644 53BP1 foci assays 1 h and 24 h after 8 Gy IR. At 1 h after IR γH2AX- or 53BP1-positive foci were counted in 15 cells per condition in non-IR cells and 24 h after IR, foci in 50 cells were counted per condition. The experiment was performed 2 times in biological replicates and results were pooled with overall *n* ≥ 15. Statistics: Two-way ANOVA with Tukey’s multiple comparisons test. Error bars are SEM. ns = not significant; * *p* < 0.05; *** *p* < 0.001; **** *p* < 0.0001 against respective non-IR cells or as indicated

Since BRAT1 expression was previously shown to be increased in radioresistant GBM cells and GSCs [18], we next examined the DNA repair proficiency in BRAT1 KD cells using γH2AX and 53BP1 foci assays. For this, we irradiated the cultures with 10 Gy (Gy) (U251 GBM cells) and 8 Gy (NCH644 GSCs) and could show different sensitivities in the response of the cell lines to irradiation (IR). In preliminary experiments we already had observed different sensitivities of the cell lines to IR. U251 shBRAT1 cells displayed a delayed DDR at both 10 Gy and 20 Gy compared to control cells (data not shown). Similarly, NCH644 shBRAT1 cells exhibited the same delayed DNA repair response at 8 Gy and 10 Gy (data not shown). Notably, lower radiation dosages produced equivalent delayed DNA repair efficacy as higher doses in both cell lines. Consequently, we selected the lower dosages for each cell line to better simulate clinical conditions. At 1 hour (h) and 24 h after IR, the cells were fixed and immunostained for γH2AX and 53BP1 to visualize and quantify DNA double strand breaks (DSBs) (Fig. 2). The same approach was performed in BRAT1 overexpressing cells (Suppl. Figure 1).

After, 1 h of 10 Gy IR, both U251 shCtrl and U251 shBRAT1 cells showed similar numbers of DNA DSBs of around 30–40 foci/cell (Fig. 2A). However, at 24 h after IR, the U251 shCtrl cells displayed approximately 10 foci/cell, whereas the U251 shBRAT1 cells indicated significantly higher DNA DSB numbers of around 25 γH2AX foci/cell. These findings were confirmed by the 53BP1 staining with 10 foci (shCtrl) and 22 foci (shBRAT1), respectively (Fig. 2B). To further validate our observation, we tested the same setup on the GSC line NCH644 (γH2AX: Figures 2C and 53BP1: Fig. 2D) resulting comparable increased residual foci numbers upon BRAT1 KD. To further test our hypothesis that BRAT1 is needed for efficient DNA repair, we implemented the same setup of γH2AX and 53BP1 staining in U251 and NCH644 cells transduced with V-lentiCMV-Puro (empty vector control) and V-lentiCMV-hBRAT1-Puro for establishing BRAT1 overexpression (OE) cells. BRAT1 OE was validated using western blot analyses (Suppl. Figure 1 A). This was confirmed by densitometric quantification as well as using quantitative real-time polymerase chain reaction (RT–PCR). In both experimental setups an increase of BRAT1 protein/mRNA expression was observed, validating the function of the constructs (Suppl. Figure 1B). Therefore, we tested the same setup as described for Fig. 2 and could observe reversed effects in comparison to BRAT1-deficient cells, showing decreased γH2AX and 53BP1 foci/cell in GBM U251-BRAT1 OE (Suppl. Figure 1 C) and in GSC NCH644-BRAT1 OE cells (Suppl. Figure 1D), indicating a more efficient DDR.

### Knockdown of BRAT1 decreases migration in vitro and in vivo

**Fig. 3:**
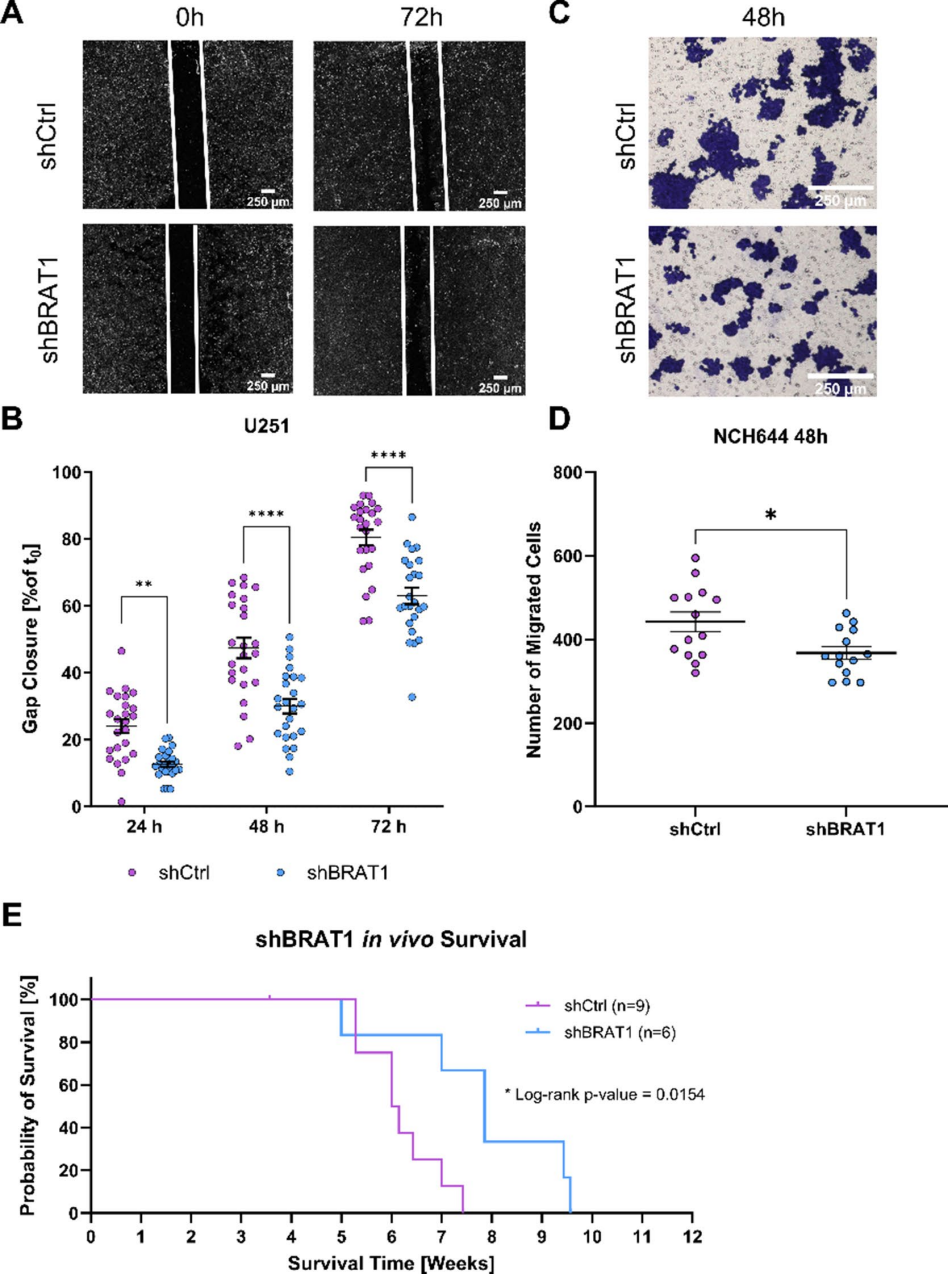
BRAT1 knockdown reduces migration in vitro and prolongs survival of NCH644 GSCs in an orthotopic transplantation model. (**A**) IBIDI migration assay pictures depicting U251 shCtrl and U251 shBRAT1 closed gap after 72 h; scale bar 250 μm (**B**) Quantification of U251 shCtrl and U251 shBRAT1 IBIDI migration assay displaying 24 h, 48 h and 72 h of the percentage of a closed gap normalized to 0 h. The experiment was performed 3 times in biological replicates, results were pooled with an overall *n* = 8 (**C**) Transwell migration assay images depict NCH644 shCtrl and NCH644 shBRAT1 fixed after 48 h; scale bar = 250 μm. (**D**) Quantification (number of migrated cells) of the transwell migration assay at 48 h. The experiment was performed 3 times in biological replicates, results were pooled with an overall *n* ≥ 4. (**E**) Kaplan-Meyer survival curve of tumor bearing mice. Mice were orthotopically implanted with either NCH644 shCtrl (*n* = 9 mice) or NCH644 shBRAT1 (*n* = 6 mice), observed over the course of 12 weeks. Statistics: B, D: Unpaired two-tailed t-test with Mann–Whitney test. E: Survival was assessed with Wilcoxon signed log rank test. Error bars are SEM, * *p* < 0.05; ** *p* < 0.01; **** *p* < 0.0001 against respective shCtrl cells or as indicated

To analyze the impact of BRAT1 expression on the migration potential in a cell-based model, we performed a two-dimensional (2D) wound healing assay to analyze the relative gap closure of U251 GBM cells and performed a three-dimensional (3D) transwell migration assay for NCH644 GSC. Important to note is the fact that GSCs can be used to investigate the same cellular activity (i.e. cell migration), but not by using the exact same methods. Since U251 (GBM) and NCH644 (GSC) cell lines not only differ in their genomic pattern, but also in their morphology type, the culturing conditions for the in vitro assays have to be adapted accordingly [9]. U251 can be cultured as a monolayer culture and can be used for 2D cell assays like the wound healing assay, here used IBDI migration assay. However, this is not possible for the NCH644 as a GSC line, since they grow in a 3D spheroidal shape. Therefore, the assay was adapted to the morphology of the cell line, in which we used the 3D transwell migration assay for NCH644 GSCs. However, the functional readout demonstrating anti-migratory effects after BRAT1 depletion remains the same. In addition, we used an orthotopic GBM mouse model to confirm the essential role of BRAT1 in promoting tumor survival (Fig. 3). First, to assess in vitro migration using a classical 2D wound healing assay, U251 shCtrl and shBRAT1 cells were seeded in IBIDI inserts, creating a defined 500 μm gap in between two adherent colonies. This allowed the monitoring of the migration capacities over the incubation time and analyzing the distance in µm between the colonies after 24 h, 48 h and 72 h (Fig. 3A and Suppl. Figure 2 A). To determine the relative gap closure, we compared the final gap width to the initial width and calculated the percentage of the closed gap. After 72 h of incubation, U251 shBRAT1 cells displayed a closed gap up to 60% compared to U251 shCtrl with an approximately 80% closed gap (Fig. 3B). Due to the 3D spheroidal morphology of GSCs, we opted for the transwell migration assay for the NCH644 shCtrl and NCH644 shBRAT1 cells. A transwell migration assay evaluates cell migration through a porous membrane towards a chemoattractant by counting migrated cells after fixation with paraformaldehyde and staining with crystal violet. A similar assay is using Boyden chamber assay, where cells invade the matrigel of the chambers [33]. Overall, the validation of the IBIDI and transwell migration assay is different, nonetheless the main readout observing the overall migration potential after BRAT1 depletion remains the same. Therefore, similar findings were observed in NCH644 BRAT1-depleted cells fixed after 48 h incubation (Fig. 3C), with around 400 migrated cells compared to NCH644 shCtrl, with approximately 440 migrated cells (Fig. 3D). Taken together, that in both cell lines the migration potential is decreased after BRAT1 depletion. To further asses the in vivo relevance and translation, we implanted NCH644 shBRAT1, as well as BRAT1-expressing NCH644 shCtrl into athymic nude mice and observed disease development (scores and body weights) and mouse survival over 12 weeks. We opted for the NCH644 GSC line for our in vivo assay, since it represents a more authentic GBM in vivo model that more closely resembles the tumor setting of this disease [34]. Therefore, BRAT1-depletion significantly enhanced median overall survival from 42.5 days (d) of shCtrl NCH644 tumor-bearing mice to 55 d, indicating that BRAT1 depletion prolonged survival (Fig. 3E). While U251 cells are perhaps better suited to investigate basal biological aspects in vitro and to further test the robustness of our results, we performed another IBIDI migration assay, using U251 empty vector control cells and U251 BRAT1 OE cells (Suppl. Figure 2B and 2 C), where a reversed effect compared to BRAT1 depletion was observed. By this, the gap was closed faster in BRAT1 OE cells after 72 h incubation compared to the control (Suppl. Figure 2B). This was further confirmed by quantification, that BRAT1 OE led to a 90 to 100% closed gap, compared to empty vector controls with a 60 to 80% closed gap after 72 h incubation (Suppl. Figure 2 C).

In addition, we assessed if BRAT1 influenced the stemness potential of GSCs, and therefore analysed the ability to form spheres in suspension cultures [35]. For this purpose, we used the GSC line NCH644. In that context, shCtrl versus shBRAT1 and empty vector control cells versus BRAT1 OE cells were implemented. After incubation for 7 d the cells were analyzed with a Tecan Spark plate reader and sphere number (Suppl. Figure 3A) and sphere area (Suppl. Figure 3B) was determined using Fiji ImageJ software [36]. As given in supplemental Fig. 3A, BRAT1 depletion leads to less sphere numbers (~ 20 spheres) compared to shCtrl cells (~ 70 spheres), whereas BRAT1 OE (~ 100 spheres), leads to an increased sphere number compared to empty vector control cell line (~ 70 spheres). Interestingly, the sphere area is only significantly reduced in BRAT1-depleted cells (~ 600 μm) compared to shCtrl (~ 800 μm), whereas BRAT1 OE (~ 710 μm) only displays a trend influencing the sphere area compared to its empty vector control (~ 700 μm) by having slightly bigger spheres, likely because of the already robust endogenous expression level of BRAT1 (Suppl. Figure 3B).

### Global proteomic- and phosphoproteomic data reveals that proteins related to migration and invasion are downregulated upon BRAT1 knockdown

**Fig. 4:**
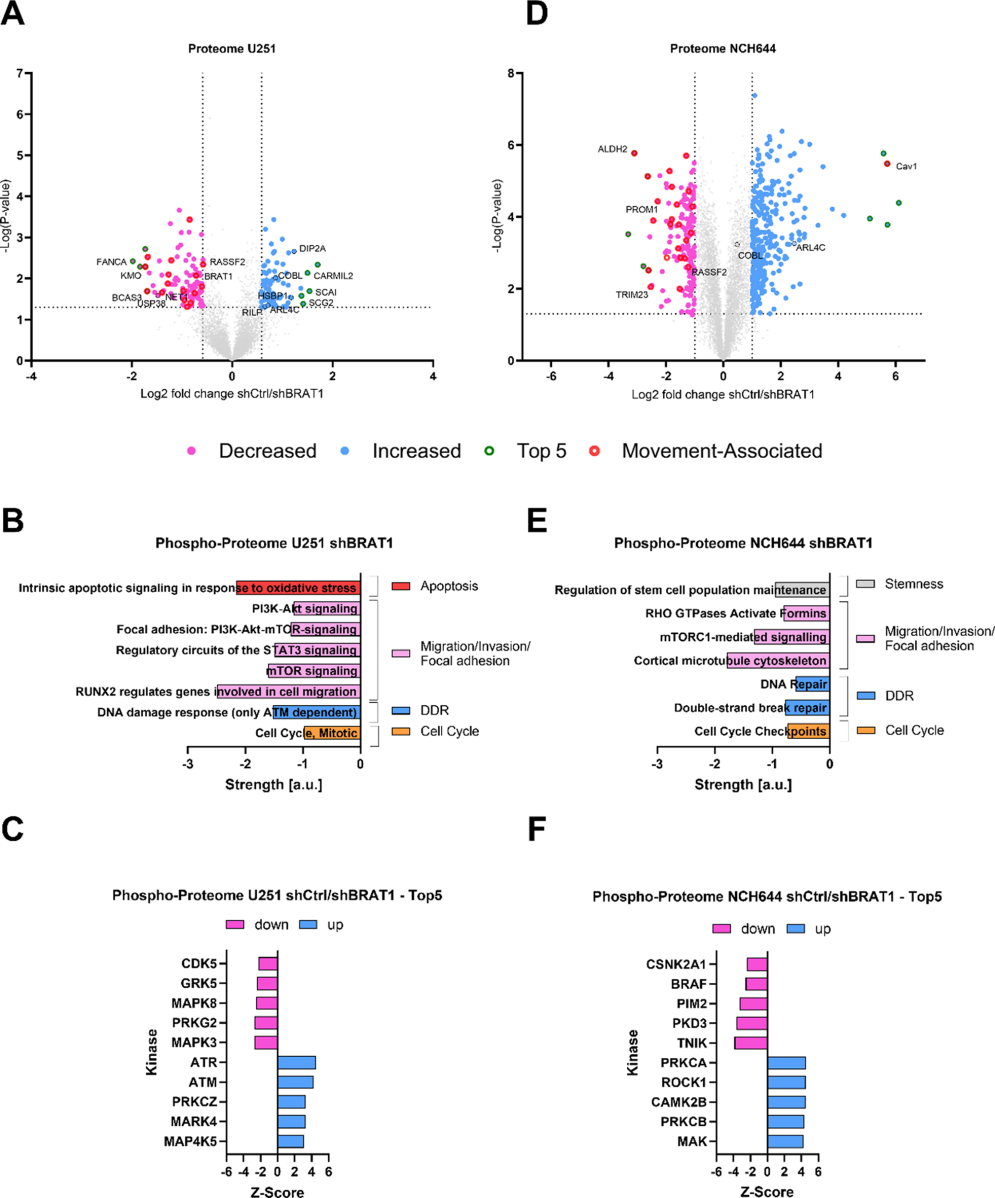
Proteomic and phosphoproteomic analysis suggest that BRAT1 influences migration and invasion in U251 GBM cells and NCH644 GSCs. (**A**) Volcano Plots of U251 shCtrl/shBRAT1 proteomic data show significantly decreased (pink) or increased (blue), the top 5 most strongly (green dots) changed proteins upon BRAT1 KD and proteins related to “movement” are depicted in red dots. (**B**) Pathway enrichment of decreased proteins of phosphoproteomic dataset using STRING software in U251 cells after BRAT1-depletion, depicted in overall strength. Decreased pathways were associated with the terms: apoptosis, cell migration/invasion/focal adhesion, DDR and cell cycle. (**C**) Kinase-Substrate-Enrichment-Analysis (KSEA) of U251 upon BRAT1 depletion phosphoproteomic data displayed in z-scores. Top5 hits suggest a loss of migratory potential and differential regulation of MAPK-pathway in BRAT1-depleted cells. (**D**) Volcano Plots of NCH644 shCtrl/shBRAT1 proteomic data show significantly decreased (pink) or increased (blue), the top 5 most strongly (green dots) changed proteins upon BRAT1 KD and proteins related to “movement” in are depicted in red dots. (**E**) Pathway enrichment of decreased proteins of phosphoproteomic dataset using STRING software in NCH644 cells after BRAT1-depletion, depicted in overall strength. Decreased pathways were associated stemness maintenance, cell migration/invasion/focal adhesion, DDR and cell cycle. (**F**) KSEA of NCH644 upon BRAT1 depletion phosphoproteomic data displayed in z-scores. Top5 hits suggest a loss of migratory potential and differential regulation of MAPK- and as well Wnt signaling pathways in BRAT1-depleted cells

In order to gain insights into the role of BRAT1 in GBM cells and GSCs we next performed a proteomic and phosphoproteomic analysis of BRAT1-depleted cells. Firstly, the global total proteome of BRAT1-depleted cells indicates changes in proteins associated with cancer cell migration and invasion in line with the findings from our previous experiments. Our proteome analysis of U251 BRAT1-depleted cells revealed a total of 7643 proteins, with 77 proteins to be significantly increased and 96 to be significantly downregulated (including BRAT1) in comparison to shCtrl cells, using distinct cut offs at *p* ≤ 0.05 and log-fold changes greater than 0.5 (Fig. 4A). Among the most strongly decreased candidates, we observed proteins related to DNA repair (FANCA, USP38, NET1), autophagy (BCAS3), cancer cell migration and/or invasion (RASSF2, KMO, BCAS3), as well as proteins that can be generally considered as pro-tumorigenic, particularly in glioma (KMO, BCAS3) (Fig. 4A). Exemplarily, Ras association domain family member 2 (RASSF2), implicated in various signaling pathways including the Hippo signaling pathway or Ras signaling pathway, which regulate cell migration and invasion, was downregulated up to 2.3-fold [37]. On the other hand, the increased candidates included proteins involved in the blockade of cancer cell migration and invasion (SCAI, CARMIL2), autophagy activation (HSBP1, RILP) as well as proteins that can be considered as anti-tumorigenic (SCG2, DIP2A, HSBP1) (Fig. 4A). Overall, these data suggest that depletion of BRAT1 evokes changes that go beyond the DDR and include changes in migratory and invasive capacity and autophagy.

Using STRING software [38] a selection of decreased pathways after BRAT1-depletion in U251 GBM cells from the phosphoproteome data was performed (Fig. 4B). Using this, BRAT1 can be possibly associated by negatively influencing pathways associated with migration/invasion and focal adhesion like: mTOR signaling, signal transducer and activator of transcription 3 (STAT3) signaling pathways and focal adhesion using PI3K-Akt-mTOR-signaling pathway. Besides this, BRAT1 may influence pathways associated to apoptosis and cell cycle.

To further confirm the findings from our STRING analysis, in which BRAT1 depletion effects the migration of cells, we analysed from the phosphoproteome dataset the Top5 suppressed (pink) and enhanced (blue) kinases upon BRAT1 depletion (Fig. 4C). Further, using the Perseus software for the phosphoproteomic data set of U251 GBM cells after BRAT1 depletion, a kinase-substrate enrichment analyses (KSEA) was performed. Therefore, observing the estimating changes of a kinase’s activity based on the collective phosphorylation changes of its identified substrates displayed in a z-score. This means, that a negative z-value depicts a kinase, which is considered downregulated upon BRAT1 depletion, because its signaling output is decreased compared to the shCtrl U251 cells [39]. Kinase‐substrate predictions were inferred from phosphoproteomic data sets of U251 GBM cells after BRAT1 depletion using the KSEA‐App (https://casecpb.shinyapps.io/ksea/). Kinase‐substrate searches included PhosphoSitePlus and NetworKIN with scores 2 or greater [39]. The phosphoproteomic content of BRAT1-depleted cells pointed towards a role for sustained DDR, autophagy and migration. After bioinformatic analyses of phosphoproteomic data of U251 cells upon BRAT1-depletion, the two most enriched kinases were ATM and ATR, as well as DNA-dependent protein kinases (DNA-PKcs), which would suggest a baseline activation of the DDR. However, the most suppressed kinases with z-scores − 2, were MAPK3, also known as ERK1 and MAPK8, also known as JNK1, which are both members of the MAPK signaling pathway, which regulates various cellular processes including cell migration and invasion [40]. Moreover, Cyclin-Dependent Kinase 5 (CDK5), involved in cytoskeletal dynamics, cancer cell migration and invasion was among the suppressed kinases [41–43] (Fig. 4C). Therefore, proteins regulating MAP2K/MAPK-signaling and cell cycle-associated processes were suppressed, suggesting a loss of a pro-migratory phenotype [43, 44].

The same analysis pipeline was applied for the GSC line NCH644 upon BRAT1-depletion, as described for the U251 dataset [39] (Fig. 4D-F). The total global proteome of NCH644 upon BRAT1 depletion (7273 proteins) suggested that 10% of proteins, which were downregulated (total 179 decreased proteins) are involved in movement, therefore potentially influencing cancer cell migration and invasion. Under the chosen parameters for data analysis, BRAT1 was not found among the most significantly downregulated proteins in the NCH644 shBRAT1 data set, despite the fact that we observed a robust depletion of BRAT1 in these cells by western blot and RT-qPCR analysis. Nonetheless, the top downregulated protein was aldehyde dehydrogenase 1 (ALDH1), which has been implicated in cancer stem cell maintenance and drug resistance and is associated with tumor invasion and metastasis (Fig. 4D) [45]. A similar function displays another protein Prominin 1 (PROM1), which was also amongst the most decreased proteins [46]. Another decreased protein was the tripartite motif containing 23 (TRIM23), which contributes to carcinogenesis in colorectal cancer and act as a poor prognostic factor [47]. Finally, RASSF2 was amongst the most decreased proteins, implying the importance of BRAT1 potentially influencing cancer cell invasion. Interestingly and further supporting our findings from the U251 proteome analysis, Caveolin-1 (Cav1) was one of the most upregulated proteins. Cav-1 has been reported to have complex effects on cancer cell migration and invasion, with both pro- and anti-migratory functions reported in different cancer types [48] (Fig. 4D).

Using STRING software [38], we analyzed and selected the pathways, that were reduced following BRAT1 depletion in NCH644 GSCs, based on the phosphoproteome data (Fig. 4E). Importantly, we could detect decreased pathway associated to stemness maintenance. This supports the findings from our stemness assay, in which BRAT1 KD/OE influences stemness capacity. Moreover, mTORC1-mediated signaling and RHO GTPases/ Activate Formins signaling pathways were decreased after BRAT1 depletion, supporting the role of BRAT1 negatively regulating migration and invasion and further validating the findings of the proteome data similar to the chemoproteomics of Cui et al. [24]. Similar to the findings of U251, pathways like DDR, including efficient DNA double-strand repair and cell cycle, were as well affected in NCH644 cells after BRAT1 depletion.

Lastly, to analyze the phosphoproteomic changes of NCH644 upon BRAT1-depletion the Top5 hits of the most significantly suppressed and enhanced kinases were selected (Fig. 4F). Among the most enhanced kinases was PRKCA, which regulates cell growth and survival, acting as both tumor suppressor and promoter depending on the physiological context [49]. Similarly, Rho-Kinase 1 (ROCK1) was enhanced, controlling cytoskeletal organization, cell movement, and matrix remodeling [50]. The most significantly suppressed kinases were associated with Wnt and MAPK pathways. Traf2 and Nck-interacting kinase (TNIK), the most suppressed kinase (z-score > -3), activates Wnt target genes and controls cell proliferation [51, 52]. Similarly, the oncogene BRAF, part of the MAPK/ERK pathway, and Protein kinase D3 (PKD3), which promotes metastasis through ERK-STAT1/3-EMT signaling, were also notably suppressed [53–57].

Under the chosen parameters of data analysis, BRAT1 was found among the most decreased proteins in our U251 dataset, but not in our NCH644 dataset, comparing shCtrl vs. shBRAT1. Candidate RASSF2 was found in both decreased proteome protein populations comparing U251 and NCH644 after BRAT1 depletion, indicating a influence on migrative/invasive capacities. After BRAT1 depletion, two hits were upregulated in both U251 and NCH644 proteome datasets: ADP-ribosylation factor-like 4 C (ARL4C) and Cordon-bleu WH2 repeat protein (COBL). ARL4C is frequently overexpressed in various cancers due to aberrant activation of Wnt–β-catenin and EGF–RAS pathways [58]. COBL, an actin nucleator, plays a crucial role in cellular morphogenesis, particularly in neuronal development [59]. All in all, after analysis of the proteome data sets, we noticed only a small overlap comparing target candidates from all the results. Therefore, we hypothesize that BRAT1 displays broader common effects on the biological processes level represented in our global proteome and phosphoproteomic analysis, rather than at the level of individual candidate proteins. All indicated proteins and their functions are listed in supplemental Fig. 4 for better oversight.

### Curcusone D, a BRAT1 inhibitor, decreases the pro-migratory/invasive phenotype of GBM and synergizes with irradiation

A study in 2021 characterized new curcusone diterpenes, which have shown promising anticancer properties and interestingly BRAT1 was identified as a key cellular target of the curcusones using chemoproteomics [24]. Based on these findings, CurD served as a pharmacological tool to inhibit BRAT1 in this study. We analyzed whether CurD could mimic the effects of BRAT1 silencing in our BRAT1 KD cells, and whether it may exert any off-target effects. First, to test the robustness of our readout we opted to check two additional cell lines, one being the 2D GBM cell line MZ-54 [60] and another 3D spheroid GSC cell line GS-5 [61], which both exhibit relatively high *BRAT1* expression (Suppl. Figure 5 A). Therefore, various migration in vitro assays were performed on a variety of GBM/GSCs lines, analyzing the growth-inhibitory effects evoked by BRAT1 inhibition, but remaining the same readout of the influence on the migration potential after CurD treatment (Suppl. Figure 5B-D). By using wound healing IBIDI assays, we could confirm that 3 µM CurD time-dependently blocks migration of 2D cell cultures using the established GBM cell line U251 (Suppl. Figure 5B), similarly as genetic BRAT1-depletion. Indeed, treatment of MZ-54 cells with 3 µM CurD evoked similar anti-migratory effects compared to U251 cells (Suppl. Figure 5B). Further, migration potential of NCH644 GSCs was observed using the transwell migration assay, in which CurD treatment reduced the number of migrated cells after 48 h (Suppl. Figure 5 C). To further validate our findings, we tested the migration potential of the 3D spheroid GSC line GS-5 [61] using a sphere migration assay [26, 61]. GS-5 similarly to NCH644 express marker proteins associated with stemness [62]. Using a tumor sphere migration assay, we observed that CurD treatment inhibited the migration potential of GBM spheres (Suppl. Figure 5D). However, the specific assay had to be adapted for the different GSC lines based on their different growth properties. Despite these technical variations, the overall functional readout demonstrated the anti-migratory effects of CurD across both adherent 2D GBM and 3D GSC spheroid models remained the same, supporting the pro-migratory role of BRAT1 in GBM.

We next validated that CurD leads to enhanced numbers of DSBs and measured γH2AX fluorescence intensity via flow cytometry in both U251 GBM cells and NCH644 GSCs (Suppl. Figure 5E). In U251 GBM cells (Suppl. Figure 5E, left panel), 24 h treatment with CurD mildly increased the amount of γH2AX even in the absence of radiation. Shortly after 10 Gy IR (1 h) the fluorescence intensity was significantly increased and further enhanced under CurD-treatment. 24 h post-10 Gy IR the γH2AX fluorescence intensity had recovered to baseline values in DMSO-treated U251 GBM cells, whereas treatment with CurD still had significantly increased DSB fluorescence signal. Similar results were obtained in NCH644 GSCs (Suppl. Figure 5E, right panel), although at 24 h post-8 Gy IR the remaining amount of γH2AX fluorescence intensity was only slightly higher compared to DMSO-treated GSCs.

**Fig. 5:**
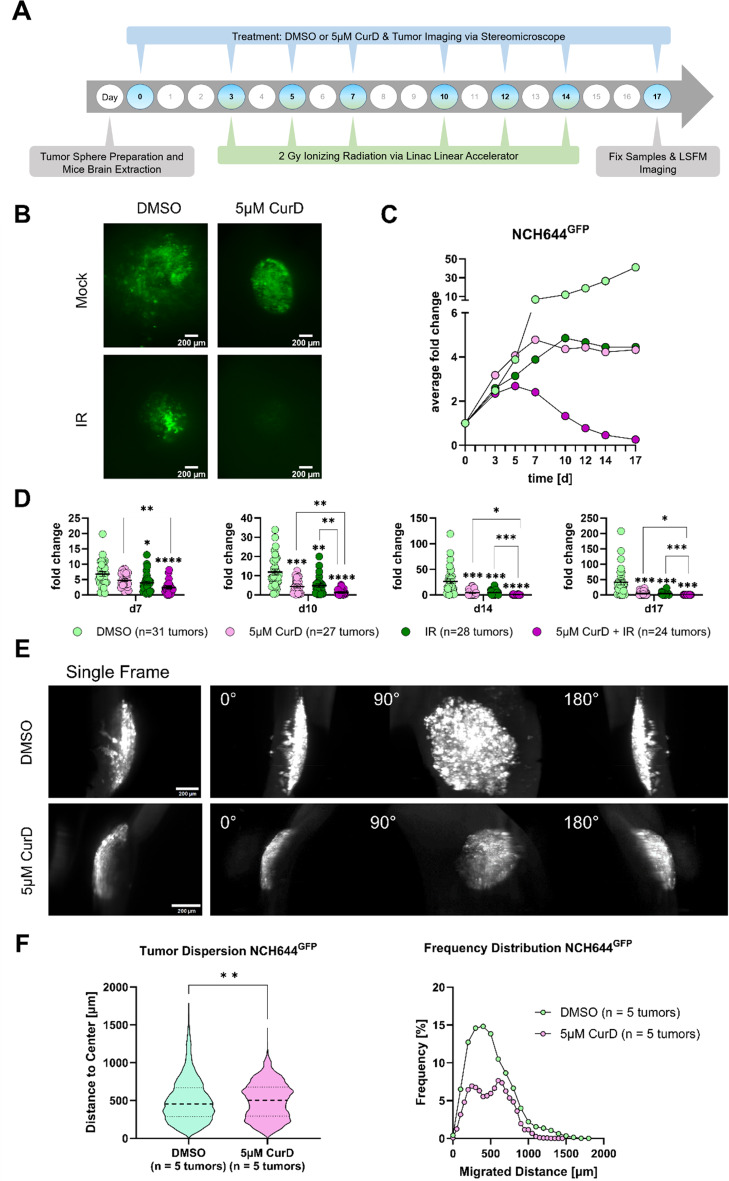
BRAT1 inhibitor Curcusone D inhibits tissue invasion and migration in an ex vivo OTC model and synergizes with irradiation treatment. (**A**) Treatment and radiation protocol as a schematic timeframe of the OTC experiment. NCH644^GFP^ were used in this experimental setup and were treated with DMSO, 5 µM CurD monotherapy or fractionated radiation therapy with 3 fractions of 2 Gy for two weeks and in combination of 5 µM CurD and fractionated IR. The culture medium containing CurD treatment was renewed on the day of radiation 2–3 h prior IR. (**B**) Representative stereomicroscopic tumor images of GSC line NCH644^GFP^ after treatment on d17. Scale bar = 200 μm. (**C**) Averaged growth kinetic throughout the observation time (timeline) of NCH644^GFP^ cells treated with DMSO as solvent control (*n* = 31 tumors), 5 µM CurD (*n* = 27 tumors), IR (*n* = 28 tumors) and combined treatment (5 µM CurD + IR: *n* = 24 tumors). (**D**) Scatter plots show tumor growth at specific timepoints (d7, d10, d14 and d17) of the experiment shown in C. (**E**) The montage of 3D reconstruction LSFM images show the same tumor at the latest time point (d17) of incubation for NCH644^GFP^ treated with DMSO or 5 µM CurD displayed in a single frame and 90° angles. Scale bar = 200 μm. (**F**) Evaluation of the LSFM images for NCH644^GFP^ at the latest time point (d17). Violin plot depicting tumor dispersion (left panel) and frequency distribution (right panel) showing the relative amount of migration distance in 500 μm bins of DMSO (*n* = 5 tumors) and 5 µM CurD (*n* = 5 tumors), all being biological replicates. Statistics: D: Two-way ANOVA with Tukey’s multiple comparisons test. F: Unpaired t-test. Error bars are SEM. * *p* < 0.05; ** *p* < 0.01; *** *p* < 0.001; **** *p* < 0.0001 against respective DMSO treated cells or as indicated

Based on the above-presented data we inferred that (1) BRAT1-depletion might hinder tumor growth in more complex model systems and (2) BRAT1-depletion might also negatively impact tumor cell migration/invasion. In order to address both question, we used an ex vivo approach consisting of organotypic tissue slice cultures (OTCs) of adult murine brains unto which GFP-positive tumor spheres are transplanted [18, 26, 63, 64]. Fluorescently labeled tumor cells, in this case NCH644^GFP^ GSCs [26], were placed onto murine brain slices and tumor development up to 17 d was monitored. We also opted again for NCH644 GSCs, since we implemented these cells in our in vivo study, as well by representing a more authentic GBM model that more closely resembles the tumor setting [34]. We applied 5 µM CurD to the established tumors on the OTCs at d0, which was followed by a fractionated radiation protocol consisting of 3 times of 2 Gy for two weeks (Fig. 5A). We opted for a higher concertation of CurD for this ex vivo assay, since this experiment displays a more complex and more translational setup of drug delivery. In more detail, the ex vivo setup uses a mouse brain slice, therefore the thickness of the slice which the compound (CurD) has to diffuse/pass physical barriers to reach the tumor cells, is far greater compared to in vitro studies, where the compound directly interferes with the cells. Additionally, the compound may be partially adsorbed while diffusing through the tissue to reach the tumor cells. Exemplary images of the tumors at d17 are displayed in Fig. 5B and additionally the intermediate images from d0, d3, d5, d7, d10, d12, d14 and d17 are displayed in supplemental Fig. 6A. Interestingly, the treatment with CurD caused a change in morphology of the GSCs compared to the control and the combination of CurD and IR resulted in an almost complete eradication of the tumor. Further, quantification of the tumor area confirmed the morphologic assessment (Fig. 5C). Solvent control (DMSO-treated) and non-irradiated tumors grew continuously over the observation period and reached a 30-fold increased size compared to d0. CurD-treatment and IR alone evoked a similar growth-suppressing effects. Lastly, the combination of CurD and IR led almost to a complete elimination of the tumors (Fig. 5C). To assess the dynamics of tumor growth of this model, tumor areas of specific timepoints were selected and displayed in Fig. 5D. Specifically, in CurD treated tumors, the tumor increased in size until approximately d7 and then reached a plateau. Hence, IR-treated tumors were significantly smaller at d7 and reached a plateau at d10. These results suggest that a combination would possibly potentiate the effects of both treatments. As such the tumors increased in size until d5 and declined in size afterwards. Strikingly at the end of the experiment (d17) 30% of the tumors (8 of 24) were completely eliminated (Fig. 5D). To highlight, irradiated tumors appeared similarly invasive to solvent-treated tumors, but with decreased sizes, however CurD-treatment led to tumors with distinctly clear-cut borders, suggesting complete failure to invade the brain tissue. To confirm this noticeable tumor morphology change, rather than tumor size, after CurD treatment, we applied an imaging technique combining OTC culture with light sheet fluorescence microscopy (LSFM) called OTCxLSFM [26] to measure the tumors and its particular single cell invasion in a 3D manner and display it in 90° angles (Fig. 5E). This elevated microscopy approach stands for a technique that combines fixed OTCs with advanced LSFM [26], in which we confirmed that DMSO-treated tumors display diffuse 3D infiltration and spreading of cells into the surrounding brain tissue (Fig. 5E). In contrast, CurD-treatment devoided tumor migration (Fig. 5E). A major advantage using this technique is the fact, that the same samples prior observed via epifluorescence microscope and therefore the samples used for quantification of tumor area can be analyzed again using LSFM after fixation and clearing of the tissue using CUBIC solution. We opted to only observe the 3D tissue invasion/migration on the last day of incubation being d17, since this had the most robust effect in our area quantification. The 90° montages of 3D reconstruction LSFM images in Fig. 5E show the same tumor and their tissue infiltration at the latest time point (d17) of incubation for NCH644 treated with DMSO or 5 µM CurD. Following this technique described by Haydo et al., we quantified and analyzed the LSFM images using Fiji and the publicly available quantification pipeline [26, 36, 65]. The quantitative assessment, using the invasion distance measured from the tumor center as a readout, confirmed the differences of tumor invasion, by decreased migration distance in CurD treated tumors with a maximal migrated distance of 1450 μm compared to solvent control cells with a maximal migrated distance of up to 1750 μm (Fig. 5F, left panel), whereas the frequency distribution of such distances revealed the overall diffusion of the cells. Both groups displayed an average migrated distance of approximately 500 μm in solvent control cells and CurD treated tumors. However, in solvent control cells about 15% met this distance, whereas only about 6% of CurD treated cells reached this distance (Fig. 5F, right panel). Our 3D approach revealed a reduction in tumor cell spreading and frequency distribution, again indicating a decreased migration potential and invasiveness by blocking BRAT1 using CurD.

Lastly, to confirm that the observed effects of CurD are not caused by any off-target effects, we performed an additional OTC experiment, where NCH644 wt^GFP^ as well NCH644 shBRAT1^GFP^ cells were treated with 5 µM CurD and subsequently observed over the same time period as described in Fig. 5 (Suppl. Figure 6B and 6 C). Indeed, we could demonstrate that no significant difference in growth behavior was observed when BRAT1-depleted tumors were treated with CurD, suggesting that major off-target effects of the drug can be excluded under the chosen conditions (Suppl. Figure 6B and 6 C).

## Discussion

GBM, the most malignant primary brain tumor in adults, is usually treated with combined RCT following surgery according to current standards, despite its pronounced radio- and chemoresistance [6]. GBM further exhibits strong pro-migratory and pro-invasive features [66, 67] and it is believed that the persistence of GSCs in this tumor is one of the major causes for treatment resistance, intra-tumoral heterogeneity and the pro-migratory/invasive phenotype. These GSCs can self-renew infinitely, upregulate DNA damage repair pathways and escape RCT by surviving in a quiescent state [15, 68]. In addition, these cells show a high capacity of cell invasion and cell migration [26]. Our previous proteomic analyses revealed that the BRCA1-interacting protein BRAT1 is one of the most prominently decreased proteins after combined in vitro treatment of GSCs with the differentiation-inducing agents ATO and Gos [18]. Similarly this treatment (ATO and Gos) inhibited tumor growth in our OTC ex vivo model, accompanied by reduced levels of proteins clustered under the term movement (40% of total decreased proteins), as well as a decrease in the levels of key DDR components [18]. These findings raised the question whether BRAT1 might play a functional role in GBM´s resistance to DNA damage and regulation of GBM migration/invasion. Of note, BRAT1 mutations are linked with neurodegenerative diseases and neurodevelopmental disorders such as rigidity and multifocal-seizure syndrome, although the exact molecular mechanisms underlying disease pathology are not well understood [69]. To highlight, recent studies have identified that BRAT1 forms a trimeric complex with the INTS11 and INTS9 subunits of the Integrator complex in human cell lines. This interaction has been shown to regulate the expression of neurodevelopmental genes by processing the 3’ ends of various non-coding RNAs and pre-mRNA [22, 23].

Furthermore, expression of BRAT1 is higher in grade 4 gliomas compared to lower-grade gliomas and positively correlated with decreased overall patient survival not only in glioma, but also in renal and liver cancer, identifying BRAT1 as a putative new target for cancer treatment (https://www.proteinatlas.org/ENSG00000106009-BRAT1/pathology). To study the functional role of BRAT1 in GBM, shRNA-mediated BRAT1 depletion was used as a strategy to investigate various aspects of cancer pathophysiology and the cellular radiation response. To address the potential relevance of BRAT1 for the DDR in GBM, the time-dependent efficiency of radiation-induced DNA repair was analysed, as BRAT1 has been described to interact with ATM and BRCA1, two fundamental proteins in the early DDR [70]. Based on the assumption that BRAT1 (1) is needed for ATM phosphorylation, (2) is needed for the function of BRCA1 and (3) interacts with DNA-PKcs, we hypothesized that DNA DSBs introduced by IR may not be repaired adequately in cells depleted of BRAT1, while OE of BRAT1 will result in a reverse effect on the DDR [19, 71, 72]. In U251 GBM cells, shCtrl and shBRAT1 cells showed equal levels of DSBs 1 h after IR with 10 Gy. However, 24 h after IR, almost all IR-induced DNA DSBs were repaired in the control cells, but significantly elevated levels of remaining DSBs were evident in BRAT1-depleted cells, indicating a defective DNA repair. This was also true in NCH644 GSCs, where we additionally observed that already 1 h after IR with 8 Gy, significantly more DSBs were not fully repaired in the cells with depleted BRAT1. Further, BRAT1 KD has been shown to decrease phosphorylation of ATM and CHK2, crucial for effective DNA repair, as well preventing the premature dephosphorylation and detachment of ATM and DNA-PKcs from DNA damage sites in HeLa cells [19]. Experiments revealed that BRAT1-depleted cells have more pATM- and 53BP1 positive foci due to impaired DNA repair, whereas BRAT1 OE cells had a more efficient DDR. Moreover, treatment with the BRAT1 inhibitor CurD increased foci numbers, suggesting that BRAT1 was essential for sustained DDR activation. Additionally, after IR pATM-positive foci were higher in BRAT1-KD and CurD-treated cells compared to controls, highlighting BRAT1’s role in maintaining long-lasting DDR. In parallel, Low et al. could show that BRAT1 is required for increasing the abundance of pATM by Nedd4 Family Interacting Protein 1 (Ndfip1) [71]. The results also suggested that BRAT1 is more crucial for sustained ATM phosphorylation in GSCs than in adherent GBM cells, indicating a potential therapeutic target for enhancing DNA repair efficacy.

To further investigate the pro-migratory phenotype of GBM and GSC lines [73], we tested in vitro migration to obtain insights into the migration capacity in relation to BRAT1 proficiency vs. deficiency/inhibition. Here we could demonstrate, that in a classical wound healing assay using the 2D GBM cell lines U251 and MZ-54, genetic BRAT1 depletion/BRAT1 inhibition via CurD leads to a decreased migratory potential compared to control cells. Of note, U251 and MZ-54 represent common adherently cultured GBM cell lines, while the 3D spheroid GSC lines NCH644 and GS-5 are used as models for the stem-like sub-population of GBM tumors, thereby covering the intra-tumoral heterogeneity of the disease [9]. The optimal experimental setup for quantifying the migratory capacity of NCH644 and GS-5 was adapted to the respective morphology and growth properties of the cell lines, using the 3D transwell migration assay for NCH644 and the sphere migration assay for GS-5. Using these two GSC models and functional readouts, we could demonstrate that our findings on the anti-migratory effect of BRAT1 depletion or BRAT1 inhibition obtained in adherent GBM cells are indeed transferable to GSCs, pointing to a functional role of BRAT1 in the aggressive growth behaviour of GBM. To correlate these observations with tumor growth in an orthotopic GBM mouse model, we opted for the NCH644 GSC line for our in vivo studies, because it better represents the authentic characteristics and microenvironment of GBM compared to other models [34]. Strikingly, BRAT1 depletion significantly prolonged overall survival of tumor-bearing mice, in line with evidence suggesting that BRAT1 is an unfavourable prognostic marker in several cancers (https://www.proteinatlas.org/ENSG00000106009-BRAT1/pathology).

The pro-migratory role of BRAT1 was further validated through global proteomic and phosphoproteomic analysis. The U251 proteomic analysis revealed decreased levels of proteins associated with migration (RASSF2, KMO) and invasion (BCAS3) upon BRAT1 KD. BRAT1 depletion in both cell lines also affected pathways linked to the DDR and cell cycle. Notably, Cav-1, which was increased in our NCH644 BRAT1 KD proteomic dataset, has been implicated in modulating migration in a context-dependent manner in different cancer types [48, 74]. In line with the observed suppressing effect of BRAT1 on its expression, Cav-1 has been described to function as a putative tumor suppressor in GBM, further highlighting the notion of targeting BRAT1 to inhibit the migrative and invasive capacity of this tumor [75]. Additionally, proteomic data obtained from the GSC line NCH644 suggests a regulatory role of BRAT1 on stemness properties of GBM with markers being decreased in BRAT1 KD cells (ALDH1, PROM1), as well as its connection to the DDR through ATM and ATR kinases, potentially further contributing to modulation of stemness [76] and radiotherapy resistance [45, 46, 77, 78]. Overall, the obtained phosphoproteomic data from both cell lines suggests BRAT1-dependent regulation of either migration-associated kinases like MAPK3, MAPK8, BRAF and PKD3, or regulators of cytoskeletal dynamics and cancer cell migration/invasion, such as CDK5 [24, 40–43, 53–56, 79, 80] or even modulating Wnt signaling via TNIK [51, 52].

Given the heterogeneity of the employed cell models, the overlap between BRAT1-dependent expression changes in the proteomic data obtained in the U251 and NCH644 cell models remained small. One interesting candidate was RASSF2, a Ras effector, which found to be downregulated in both proteome datasets, it connects RAS to various signaling pathways, potentially influencing tumor growth and progression. In addition, studies revealed it interacts with proteins involved in processes like EMT. These interactions suggest, that RASSF2 may play a crucial role in regulating multiple cellular processes essential for normal development, and its loss could disrupt critical regulatory mechanisms [81]. In addition, it can also act as a tumor suppressor in human colorectal cancers [82]. Two interesting candidates (ARL4C and COBL) found to be upregulated comparing U251 and NCH644 proteome datasets after BRAT1 depletion can be associated with regulating cell shape, motility, actin dynamics and neural development [58, 59]. Overall, our findings from the proteomic analyses suggests, that BRAT1 may affect biological processes at a higher systemic level, rather than through direct interactions with individual target proteins, similar to the findings of our phosphoproteomic data, in which different kinases effect overall MAPK signaling. Despite these limitations, processes related to migration were found to be downregulated in both BRAT1-deficient lines. In combination with the data obtained from the numerous functional assays employed in this study [wound healing assay (IBIDI), 3D transwell migration assay, in vivo orthotopic mice model and OTC ex vivo tumor growth], these findings collectively support the pro-migratory function of BRAT1 in GBM.

Since our data obtained in the genetic models had identified BRAT1 as an interesting target for GBM therapy, we wanted to further asses the therapeutic implications of these results. Therefore, we used CurD to target the hitherto “undruggable” protein BRAT1 for proteasomal degradation [24]. Similar to the findings obtained with BRAT1 KD, we observed anti-migratory effects after CurD treatment and decreased DDR efficiency after combined CurD and IR treatment in vitro. Since the bioavailability of CurD and its ability of passing the blood-brain-barrier has not been elucidated yet, we implemented our OTC transplantation model as a translational ex vivo assay and combined it with LSFM to gain insights into the effects of CurD in mouse brain tissue on a single cell level. We recently developed the combination of OTCs and LSFM as an advanced imaging technique, allowing to gather qualitative and quantitative 3D insights of cell migration/invasion [26]. Importantly, treatment with CurD enhanced the limiting effect of IR on tumor growth, indicating that combination of conventional therapy with BRAT1 inhibition may be an interesting therapeutic strategy. Moreover, single agent treatment with CurD led to pronounced morphology changes of GBM tumors as observed by the OTCxLSFM technique. Our data reveals that the pro-migratory, pro-invasive phenotype of BRAT1-proficient tumors can be effectively curtailed by CurD, with significantly reduced numbers of brain-penetrating GSCs in the tumor microenvironment following treatment. These data demonstrate a striking therapeutic effect of CurD on GSC tumor growth/invasion in our ex vivo OTC model. Importantly, we could confirm the specificity of CurD targeting BRAT1, since CurD treatment did not let to any discernible off-target effects when treating BRAT1 KD GSC tumors. Based on these findings, we identify BRAT1 as a novel and druggable therapeutic target for GBM and highlighting the potential of CurD as a putative new therapeutic compound.

In summary, we find that BRAT1 deficiency causes partial failure of the DDR and prevents GBM cells from invasion and migration. Results of KD experiments are recapitulated with CurD as a pharmacologic tool, hence suggesting that targeting BRAT1 might be a reasonable approach to reduce the radioresistance of GBM and limit invasiveness.

## Conclusion

Our study identifies BRAT1 as a critical driver of GBM pathology and the pro-migratory and -invasive phenotype. We demonstrate that BRAT1 depletion sensitizes GBM cells to radiation and inhibits tumor growth and invasion in both in vitro and in vivo models. Treatment with CurD, particularly in combination with radiation, effectively reduces tumor migration and brain tissue infiltration, suggesting BRAT1 targeting as a promising therapeutic strategy for GBM.

## Materials and methods

### Analysis of public datasets

Analysis of the Gravendeel dataset [27] was performed and plots were exported via the GlioVis portal (http://gliovis.bioinfo.cnio.es/) [28]. Differences of BRAT1 expression were compared between GBMs and healthy tissues based on histology and the Kaplan-Meier curve was derived from the Gravendeel datasets of all GBM subtypes combined with a median cut-off together with confidence intervals. The CGGA (Chinese Glioma Genome Atlas) dataset [29] was analyzed for overall BRAT1 mRNA expression in grade 2 tumors in comparison with tumor grades 3 and 4.

### Cells and cell culture

Experiments were performed with NCH644, GS-5 human GSCs [31, 61] and U251 and MZ-54 MG GBM cells [30, 60]. NCH644 and GS-5 cells were cultured in Neurobasal medium (Gibco, Darmstadt, Germany) supplemented with 1x B27 supplement, 100 U/mL Penicillin, 100 µg/mL Streptomycin, 1x GlutaMAX (all from Gibco), 20 ng/mL epidermal growth factor, 20 ng/mL fibroblast growth factor (both from Peprotech, Hamburg, Germany) and 2 µg/mL Puromycin dihydrochloride (sc-108071B, Lot # L2214, Santa Cruz Biotechnology, Inc., Heidelberg, Germany). U251 and MZ-54 cells were cultured in Dulbecco’s modified Eagle’s medium with GlutaMAX and heat-inactivated 10% fetal calf serum, 100 U/mL Penicillin, 100 µg/mL Streptomycin (all from Gibco) and 1 µg/mL Puromycin dihydrochloride (sc-108071B, Lot # L2214, Santa Cruz Biotechnology, Inc.). All cell lines were cultured at 37 °C and 5% CO_2_ and were passaged twice a week in a ratio of 1:10 or 1:20, respectively.

### Establishment of stable shRNA BRAT1 knockdown cells/ BRAT1 overexpressing cells/ GFP positive cells

Stable shRNA-mediated shBRAT1 cells (pLKO.1-puro_shBRAT1_A (N0000172594), pLKO.1-puro_shBRAT1_C (N0000172960) MISSION^®^ (Sigma-Aldrich, Darmstadt, Germany) or control cells expressing non-mammalian targeting control shRNA (pLKO.1-Puro_shCtrl; MISSION^®^ SHC002, Sigma-Aldrich) were generated using HEK293T cells. In addition, NCH644 and U251 cells were transduced with V-lentiCMV-Puro for empty vector control cells and V-lentiCMV-hBRAT1-Puro (self-generated) for producing BRAT1 OE cells. Briefly, 150,000 of HEK293T cells were seeded per well into 6-well plates the day before transfection with 2 µg plasmid DNA (pLKO.1-puro), 1.5 µg gag/pol plasmid (psPAX2, addgene #12260) and 0.5 µg VSV-G envelope plasmid (pMD2.G, addgene #12259) in 57 µL Opti-MEM (Invitrogen, Frankfurt am Main, Germany) and 6 µL FuGENE HD (Promega, Walldorf, Germany) transfection reagent. Six hours later, medium was changed, and the viral supernatant was collected after additional 16 h and 40 h, pooled and filtered through a 0.45 μm filter, followed by dilution of the supernatant with fresh medium in a ratio of 1:1 and addition of 3 µg/mL hexadimethrine bromide (polybrene, Sigma). 1 µg/ml (U251) and 2 µg/ml (NCH644) Puromycin (Sigma) was added and maintained to select for positively transduced cells. psPAX2 and pMD2.G were gifts from Didier Trono (Addgene plasmid # 12260; http//n2t.net/addgene:12260; RRID: Addgene_12260; Addgene plasmid # 12259; http//n2t.net/addgene:12259; RRID: Addgene_12259). Lastly, NCH644 shBRAT1 cells were again transduced as described above with pLV(Exp)-EGFP: T2A: Bsd-CMV > ORF_Stuffer (Vector-Builder GmbH, #VB181209-1075efx) to produce NCH644 shBRAT1^GFP^ cells used for OTCs and 2 µg/ml Puromycin and 1.25 µg/mL Blasticidin (Sigma) were added and maintained to select for positively transduced cells.

### Compounds

The BRAT1 inhibitor Curcusone D was dissolved and diluted in DMSO (Sigma) and stored at -20 °C. An intermediate dilution of 3 mM was used for all other dilutions. It was prepared and characterized by the laboratory of Mingji Dai [24].

### Irradiation and antibodies

Cells were irradiated with single doses of 2, 8–10 Gy using a linear accelerator (Elekta Synergy, Elekta, Stockholm, Sweden) with 6 MV photon energy, 100 cm focus to isocenter distance and a dose rate of 6 Gy/min while non-irradiated control cells were kept in a transportation box in parallel, providing the same experimental conditions.

The following primary antibodies and dilutions were used: mouse-anti-γH2AXSer139 1:1,000 (#05-636, clone JBW301, Merck Millipore) and rabbit-anti-53BP1 1:1,000 (NB#100–304, Novus Biologicals) for immunofluorescence; rabbit-anti-BRAT1 1:250 (HPA029455, Sigma) and mouse-anti-Tubulin 1:5,000 (T6199, Sigma) and mouse-anti-GAPDH 1: 20,000 (#MS-369-P0, Thermo Fischer) for western blot. For flow cytometry the FITC conjugated primary antibody γH2AXSer139–FITC 1:250 (16–202 A, Merck Millipore) was used.

The following secondary antibodies with corresponding dilutions were used: F(ab’)2-goat-anti-rabbit IgG (H + L) cross-adsorbed secondary antibody; Alexa Fluor 488 1:500 (A-11070, Thermo Fisher); F(ab’)2-goat-anti-mouse IgG (H + L) cross-adsorbed secondary antibody; Alexa Fluor 594 1:500 (A-11020, Thermo Fisher) for immunofluorescence. IRDye 800CW goat-anti-rabbit 1:10,000 (926-32211) and IRDye 680RD goat-anti-mouse 1:10,000 (926-68070; both LI-COR Biosciences) for western blot.

### RT-qPCR

To analyze differential gene expression in GBM and GSCs, quantitative RT–PCR was performed as previously described in Haydo et al. [26]. Shortly, RNA was isolated from 300,000 cells using the ExtractMe Total RNA Kit (7Bioscience, Hartheim, Germany; https://blirt.eu/wp-content/uploads/2018/08/TOTAL‐RNA_protocol_en_29012018_druk_v2‐min.pdf). cDNA was synthesized using standard protocols and analyzed by qRT-PCR with TaqMan probes for TBP (Hs00427620_m1) and BRAT1 (Hs01018277_m1). Gene expression was quantified using the ΔΔCT method with TBP as housekeeping gene.

### SDS PAGE and western blotting

Cell lysis, SDS-PAGE and Western Blotting were performed as previously described by Antonietti et al. [83]. In short, membranes were blocked for 1 h at room temperature with 5% BA/TBS-Tween-20 (TBS-T), followed by an incubation with the primary antibodies as detailed above diluted in 5%BSA/TBS-T at 4 °C overnight. The secondary antibodies, diluted in 5% BA/TBS-T, were incubated for 1 h at room temperature and signals were detected using a LI-COR Odyssey reader (LI-COR Biosciences). Protein expression levels were quantified using densitometric quantification. To this end, the respective protein amount of BRAT1 was first normalized to the loading control (Tubulin/GAPDH) and afterwards the different genetic models were normalized to their respective controls, that were set to 100%. Original western blots used for KD quantification are available via zenodo.org (10.5281/zenodo.14390501) [32].

### γH2AX intensity using Flow Cytometry

U251 and NCH644 cells (6 × 105 cells/mL, 0.5 mL/well) were seeded in 24-well plates. The next day, the cells were treated with 3 µM CurD for 48 h and irradiated with 8 Gy (NCH644 GSC) and 10 Gy (U251 GBM cells). Prior to the measurement, the cells were harvested and stained using γH2AXSer139–FITC 1:250 (16–202 A, Merck Millipore). Cells were analyzed with BD Accuri C6 flow-cytometer (BD Biosciences) and data processing was done using BD Accuri C6 software (BD Biosciences) extracting the mean fluorescence intensity (MFI).

### Microscopy

#### γH2AX and 53BP1 immunofluorescence Microscopy

U251 or NCH644 cells per well were seeded on 8-well chamber slides at a density of 8,000 cells/well (Falcon, Corning). For NCH644 cells, the slides were coated with 10 µg/mL laminin (Sigma, L2020) at 4 °C overnight before cell seeding. One day after seeding, the cells were irradiated as indicated. After different time points, they were fixed using 4% formaldehyde/0.25% Triton X-100/ PBS, primary antibodies were diluted in 5% BSA/PBS and incubated at 4 °C overnight. Secondary antibodies diluted in 5% BSA/PBS were incubated for 1 h at room temperature in the dark. Next, the cells were either mounted on ProLong Gold antifade reagent with DAPI (Invitrogen) or Fluoroshield with DAPI histology mounting medium (Sigma). Slides were stored at 4 °C until analysis using a Nikon Eclipse TE2000-S inverted fluorescence microscope operated by the software NIS Elements AR version 4.2 (both Nikon Instruments Europe B.V.) at 60x magnification. For the foci assay, at 1 h after IR γH2AX- or 53BP1-positive foci were counted in 15 nuclei per condition in non-IR cells and 24 h after IR foci were counted in 50 cells per condition.

#### Light-sheet microscopy

Light-sheet-fluorescence-microscopy (LSFM) was performed by established protocols by Haydo et al. to analyze the migration of NCH644^GFP^ DMSO/5 µM CurD treated cells [26, 65]. Briefly, LSFM is a fast 3D fluorescence microscopy technique. It uses a laser light sheet to selectively excite fluorescence in one plane at a time, reducing photobleaching and phototoxicity. This allows for rapid imaging of large samples with high spatial and temporal resolution. For LSFM imaging, a custom-built “monolithic digitally‐scanned light sheet microscope” (mDSLM) was used [84]. The mDSLM features a motorized xyzϑ‐stage placed below the specimen chamber. Light sheet imaging was performed with an Epiplan‐Neofluar 2.5×/0.06 illumination objective (Carl Zeiss), an N‐Achroplan 10×/0.3 detection objective (Carl Zeiss), and a Neo CCD camera (ANDOR Technology). The analysis procedures as described in Haydo et al. provides detailed 3D information at the single-cell level, enabling insights into the morphological characteristics of the tumor cells as well as infiltration or migration [26].

### Cell-based assays

#### Sphere formation

This assay was performed following the established protocols of Haydo et al. and Roth et al. [26, 35]. Briefly, to assess changes in sphere area/number and stemness after BRAT1 depletion, cells were seeded at 500 cells/well in a 96-well plate, with 5–10 replicates per condition. After incubation at 37 °C for 7 days, images were captured using a Tecan Spark plate reader (Tecan) and analyzed with Fiji ImageJ software [36] to determine mean sphere area and count.

#### Wound healing assay – IBIDI cell migration assay

To investigate the impact of the BRAT1 inhibitor CurD or BRAT1-depletion on cell migration, we employed a wound healing assay using IBIDI chambers (ibidi GmbH). IBIDI inserts offer consistent wall width (500 μm), minimizing experimental error compared to traditional scratch assays. U251 and MZ-54 GBM cells were seeded in IBIDI chambers a day prior to treatment. Measurements were taken at 0 h, 24 h, 48 h and 72 h post-treatment using a Tecan Spark reader, with gap width was quantified as percentage from baseline using Fiji ImageJ software [36] to assess migration dynamics.

#### Transwell migration

The transwell migration (cellQART^®^) assay measures the ability of cells to migrate through a porous membrane towards a chemoattractant. In this study, NCH644 GSCs were seeded (40,000 cells/chamber) in the upper chamber, and those migrating to the lower chamber are quantified after a defined period. Briefly, the transwell chamber consists of an upper chamber (insert) and a lower chamber (well). GSCs were seeded into the upper chamber of the transwell insert and the lower chamber was filled with culture medium with/without 3 µM CurD. The chambers were fixed after 48 h of incubation. Non-migrated cells on the upper surface of the membrane were removed by gentle wiping with a cotton swab and washing. Migrated cells on the lower surface of the membrane were fixed using methanol, stained with crystal violet and quantified using the Nikon Eclipse TE2000-S inverted fluorescence microscope operated by the software NIS Elements AR version 4.2 (both Nikon Instruments Europe B.V.) at 20x magnification and the number of migrated cells was quantified.

#### Sphere migration

This assay was performed following an established protocol by Haydo et al. [26]. Briefly, to analyze migration in GSC line GS-5 [61] tumor spheres, 2,000 to 3,000 cells were seeded into U-shaped 96-well plates and after 48 h transferred into conventional 96-well plates previously coated with laminin. Plates were prewarmed, and spheres allowed to adhere for 30 min. Images were taken using a Tecan Spark plate reader (Tecan) before and after 3 µM CurD and DMSO control treatment. Migration was observed up to 72 h. Images were captured every 24 h. Fiji software [36] was used to analyze images, measuring migration distance in µm from the edge of the spheres.

### Organotypic slice cultures and ex vivo tumor growth assay

For generating adult organotypic slice cultures (OTC) and perform ex vivo tumor growth assay we followed established procedures by Haydo et al., Linder et al. and Gerstmeier et al. [18, 26, 63]. Briefly, this experiment utilizes OTC, by dissecting mouse brains and generating slices into 150 μm thick slices using a vibratome. These slices were cultured on Millicell inserts (Sigma) in six-well plates with FCS-free medium. Tumor spheres were placed onto the brain slices, and treatments (5 µM CurD and 2 Gy fractioned IR) were administered every three days (Monday, Wednesday and Friday) (Fig. 5A).

### Proteomic and phospho-proteomic sample preparation and analyses

Proteomic and phosphoproteomic sample preparation and data analyses was performed by established protocols Linder et al. [85]. Briefly, the cells were grown until confluence and were lysed and the proteins were extracted. The proteins were digested using Trypsin (Promega; V5113) and LysC (WakoChemicals). After purification using SepPak C18 columns (Waters; WAT054955) the peptides were TMT-labeled (Thermo Fisher Scientific; 90061;TH266884) and fractionated with the High pH Reversed‐phase Fractionation Kit (Thermo Fisher Scientific). After HPLC mediated separation the solutions were directly sprayed into a QExactive HF mass spectrometer and the RAW data was processed with Proteome Discoverer 2.2 software (Thermo Fisher Scientific). TMTpro reporter abundances were extracted and used for plotting and statistical analysis. Further analysis was done on Perseus (v.1.6.5.0) and STRING (v. 12.0). The datasets generated and analysed during the current study are available from the corresponding author on reasonable request. The raw data files (Excel) of the proteomic and phosphoproteomic analysis containing the list of proteins/kinases and their fold change values and significance (p-value) can be obtained at zenodo.org via the link 10.5281/zenodo.14098936 [86].

### Bioinformatic analyses

We employed a combination of bioinformatical tools to analyze protein-protein interaction networks and pathway enrichment, providing insights into the functional relationships and regulatory mechanisms underlying complex biological processes, upon BRAT1 depletion. First, the STRING database [38] was utilized to explore known and predicted protein-protein interactions, allowing for the construction of comprehensive interaction networks. Additionally, the KSEA tool https://casecpb.shinyapps.io/ksea/ [39, 87–89] was utilized to investigate kinase-substrate relationships and predict potential kinase activities regulating cellular signaling pathways in our U251 and NCH644 upon BRAT1 depletion phosphoproteomic datasets. This approach provided valuable information on kinase-mediated signaling cascades and their implications in cellular physiology and disease. Observing the estimating changes of a kinase’s activity based on the collective phosphorylation changes of its identified substrates displayed in a z-score. In all, by integrating these bioinformatical tools, we were able to comprehensively analyze protein interaction networks, decipher enriched biological pathways and predict kinase activities, ultimately enhancing our understanding of the molecular mechanisms governing BRAT1 depletion.

### Animal experiments

Mouse in vivo experiments was conducted to investigate the role of BRAT1 in promoting tumor growth/invasion and its effect on overall survival using an orthotopic GBM mouse model. Athymic nude mice (*n* = 15) were used due to their lack of an innate immune system, making it possible to transplant human NCH644 shCtrl and NCH644 shBRAT1 GSC cells into the brain. The animals were housed in type II long cages, purchased from Charles River, and were kept in the “Georg-Speyer-Haus” (Frankfurt, Germany) during experiments. They had free access to food and water. All animal procedures were conducted in accordance with a valid approval (FK-1116) by the Regierungspräsidium Darmstadt and adhered to ARRIVE guidelines set out to reduce animal numbers and minimize suffering. Distinct guidelines and pain scores (score sheets) were established to observe the behavior of the mice upon tumor implantation surgery. Mice were weighted two times a week and checked every two days. If scores exceeded the defined cut off scores for pain and well-being, respective mice were euthanized using a CO_2_ chamber followed by cervical dislocation. Throughout the experiment, efforts were made to minimize the number of animals and to refine experimental procedures to reduce potential harm or discomfort.

### Statistics

All statistical analyses applied are either two-tailed, unpaired t-test for comparison of parametric and normally distributed data of two groups. If prerequisites for t-tests were not met, the non-parametric Mann–Whitney U test was used as alternative. Survival data were analysed using or Wilcoxon signed rank tests. Multiple groups were compared using one‐way ANOVA with Tukey’s or Dunnetts’s multiple‐comparison or multivariate ANOVA with Tukey’s multiple‐comparison test. Statistical analyses were done with GraphPad Prism 9 and 10 (GraphPad Software, La Jolla, CA, USA). The statistical tests are detailed in the respective figure legends.

## Electronic supplementary material

Below is the link to the electronic supplementary material.

Supplementary Material 1

Supplementary Material 2

Supplementary Material 3

Supplementary Material 4

Supplementary Material 5

Supplementary Material 6

## Data Availability

All data and materials as well as software application information are available and mentioned in the manuscript. KSEA application is available via https://casecpb.shinyapps.io/ksea/. Original western blots used for knockdown quantification are available via zenodo.org (10.5281/zenodo.14390501) [32]. The datasets generated and analysed during the current study are available from the corresponding author on reasonable request. The raw data files (Excel) of the proteomic and phosphoproteomic analysis containing the list of proteins/kinases and their fold change values and significance (p-value) can be obtained at zenodo.org via the link 10.5281/zenodo.14098936 [86].

## Abbreviations

2D: Two-dimensional
3D: Three-dimensional
ALDH1: Aldehyde Dehydrogenase 1
ARL4C: ADP-ribosylation factor-like 4 C
ATO: Arsenic Trioxide
BRAT1: BRCA1-associated ATM activator 1
Cav1: Caveolin-1
CDK5: Cyclin-Dependent Kinase 5
COBL: Cordon-bleu WH2 repeat protein
CurD: Curcusone D
D: Day
DDR: DNA Damage Response
DNA-PKcs: DNA-dependent protein kinases
DSB: Double Strand Break
EMT: Epithelial-Mesenchymal Transition
ERK: Extracellular Signal-Regulated Kinase
EV: Empty Vector
GBM: Glioblastoma
GSC: Glioma Stem-Like Cell
Gos: (-)-Gossypol
Gy: Gray
H: Hour
IR: Irradiation
INTS: Integrator Subunit complexes
KD: Knockdown
KSEA: Kinase-Substrate Enrichment Analyses
LSFM: Light Sheet Fluorescence Microscopy
MAPK: Mitogen-activated protein kinase
MFI: Mean Fluorescence Intensity
mTOR: Mechanistic Target of Rapamycin
Ndfip1: Nedd4 Family Interacting Protein 1
OE: Overexpression
OTC: Organotypic Tissue Slice Culture
PI3K: Phosphoinositide-3-Kinase
PKD3: Protein kinase D3
PROM1: Prominin 1
PRKCA: Protein kinase C alpha
RASSF2: Ras association domain family member 2
RCT: Radiation and Chemotherapy
ROCK1: Rho- kinase 1
RT-qPCR: Real-Time Polymerase Chain Reaction
STAT3: Signal Transducer and Activator of Transcription 3
TNIK: Traf2 and Nck-interacting kinase
TRIM23: Tripartite Motif Containing 23
Wnt: Wingless/integrated
