# Supplementary figures and images for: BRAT1 - a new therapeutic target for glioblastoma
Source: Cell Mol Life Sci. 2025 Jan 21;82(1):52. doi: 10.1007/s00018-024-05553-0 (PMC11747058; doi:10.1007/s00018-024-05553-0)

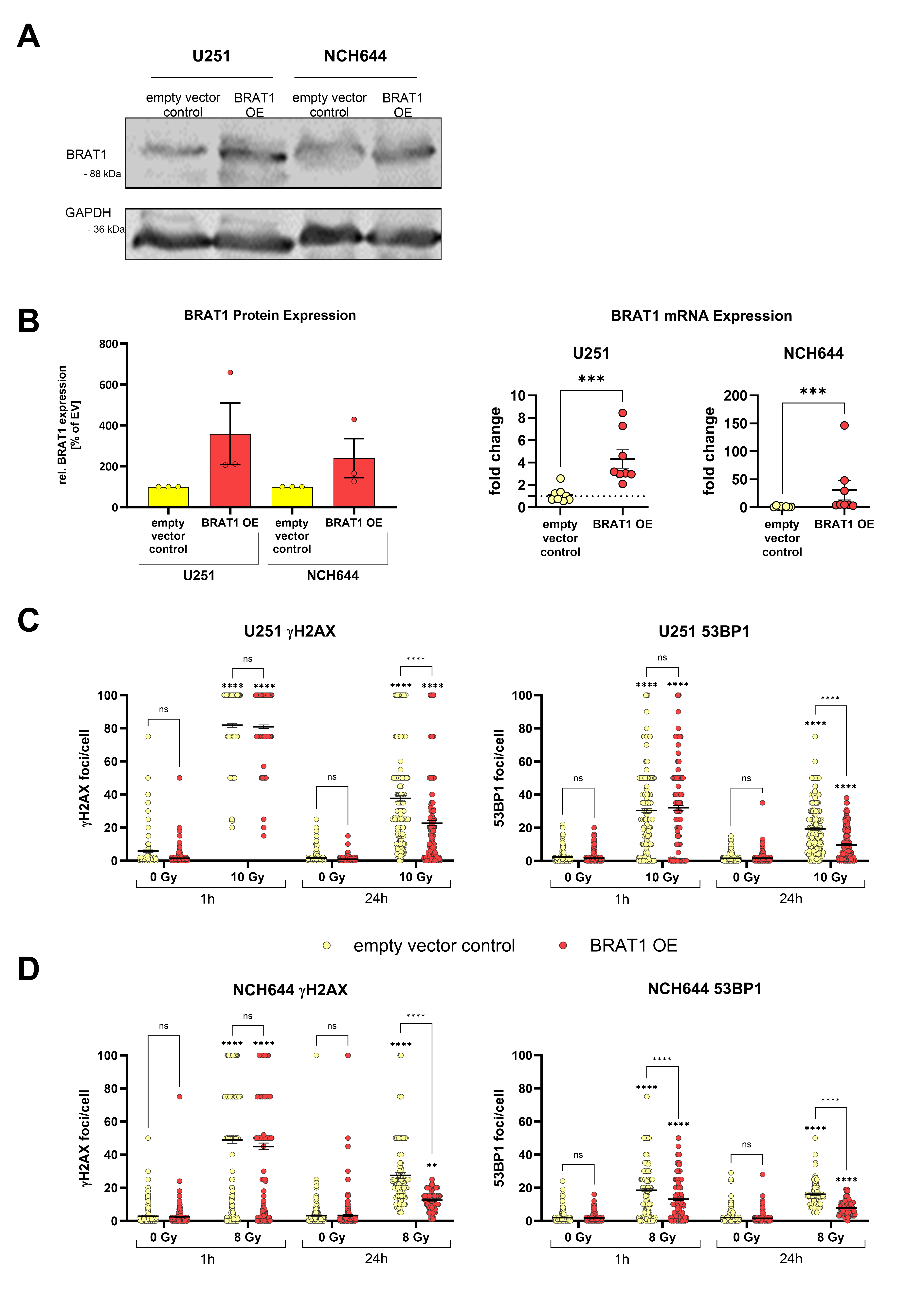

Supplement: Supplementary file 1 — Supplementary Material 1 [file 18_2024_5553_MOESM1_ESM.tif]

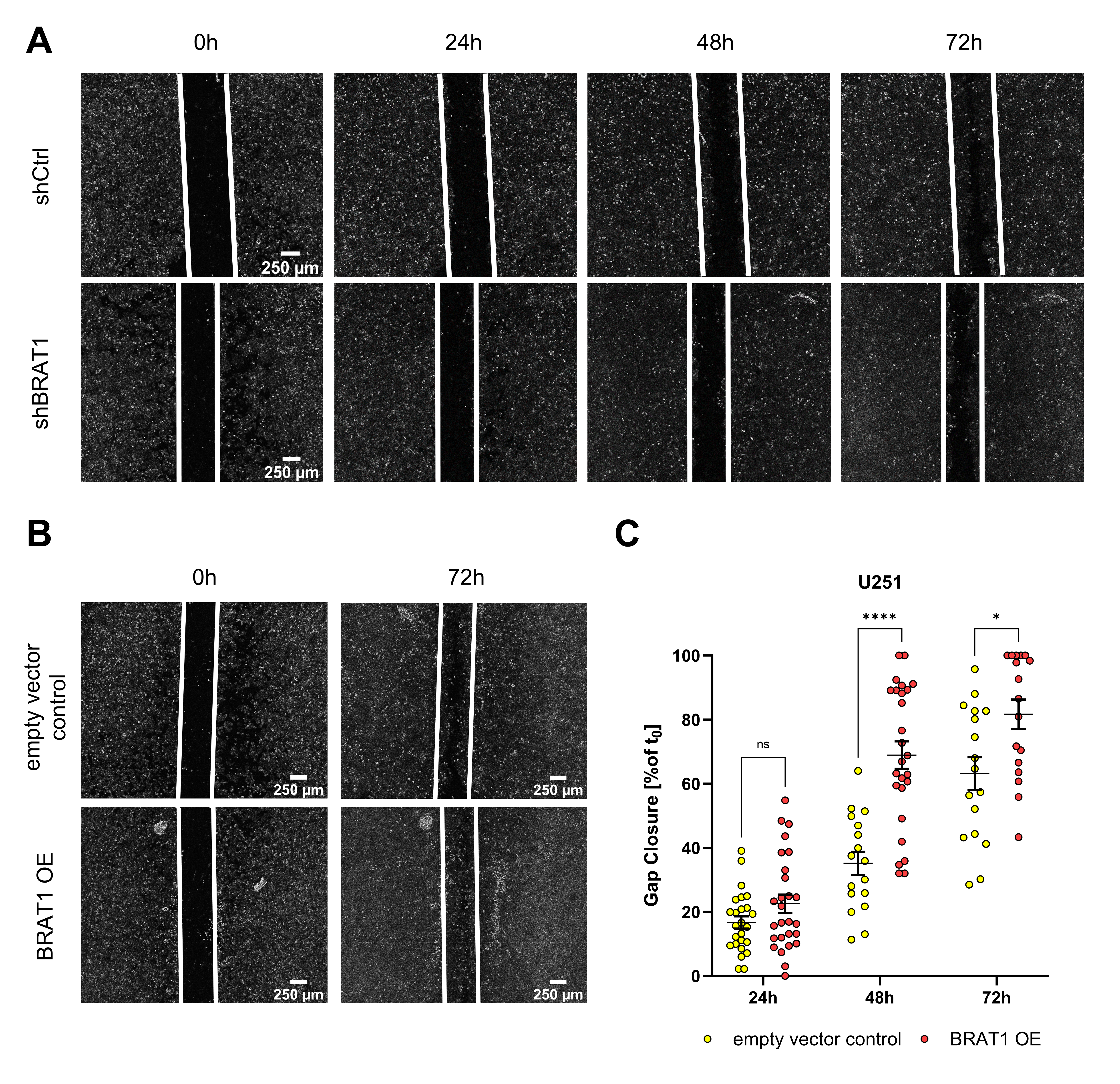

Supplement: Supplementary file 2 — Supplementary Material 2 [file 18_2024_5553_MOESM2_ESM.tif]

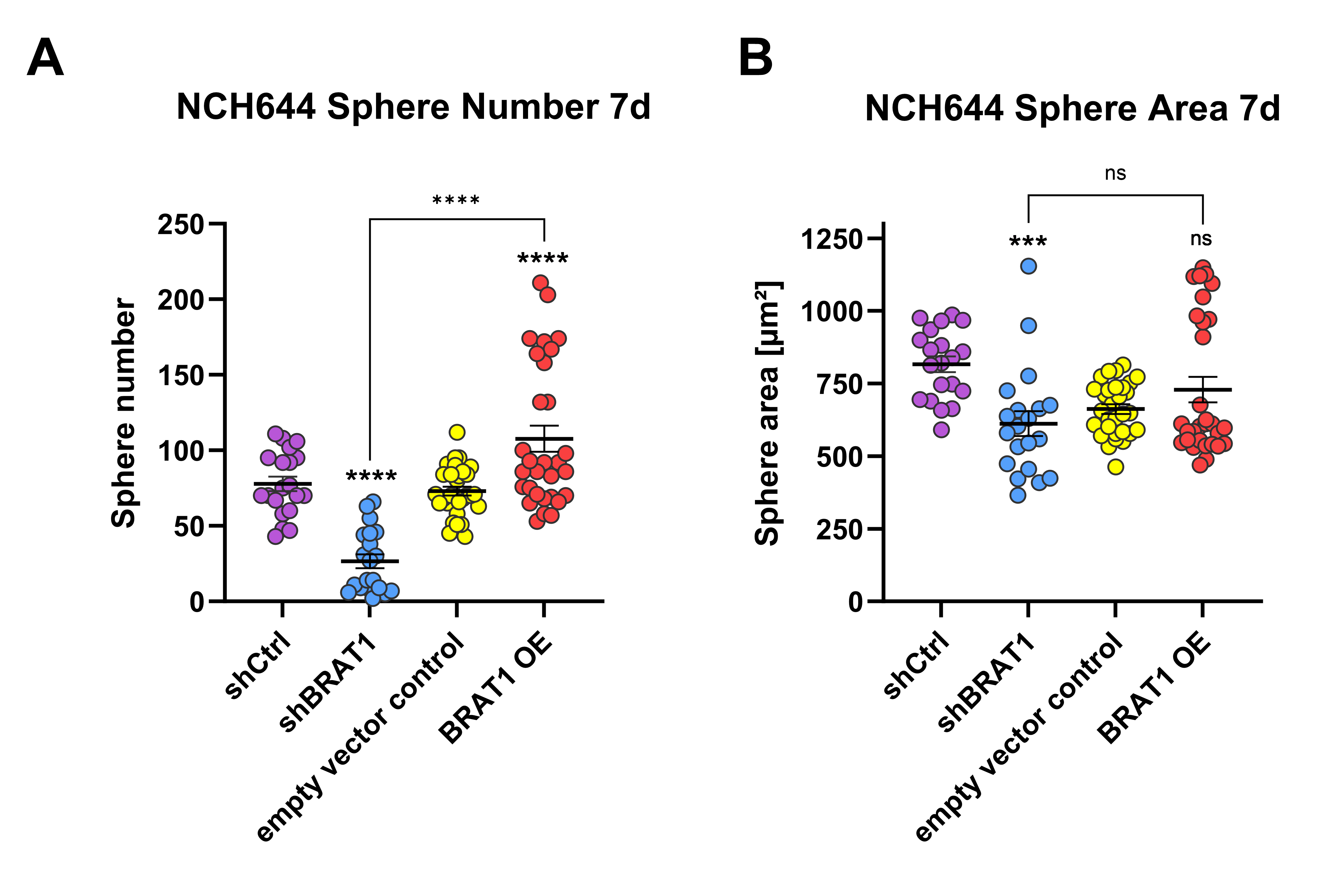

Supplement: Supplementary file 3 — Supplementary Material 3 [file 18_2024_5553_MOESM3_ESM.tif]

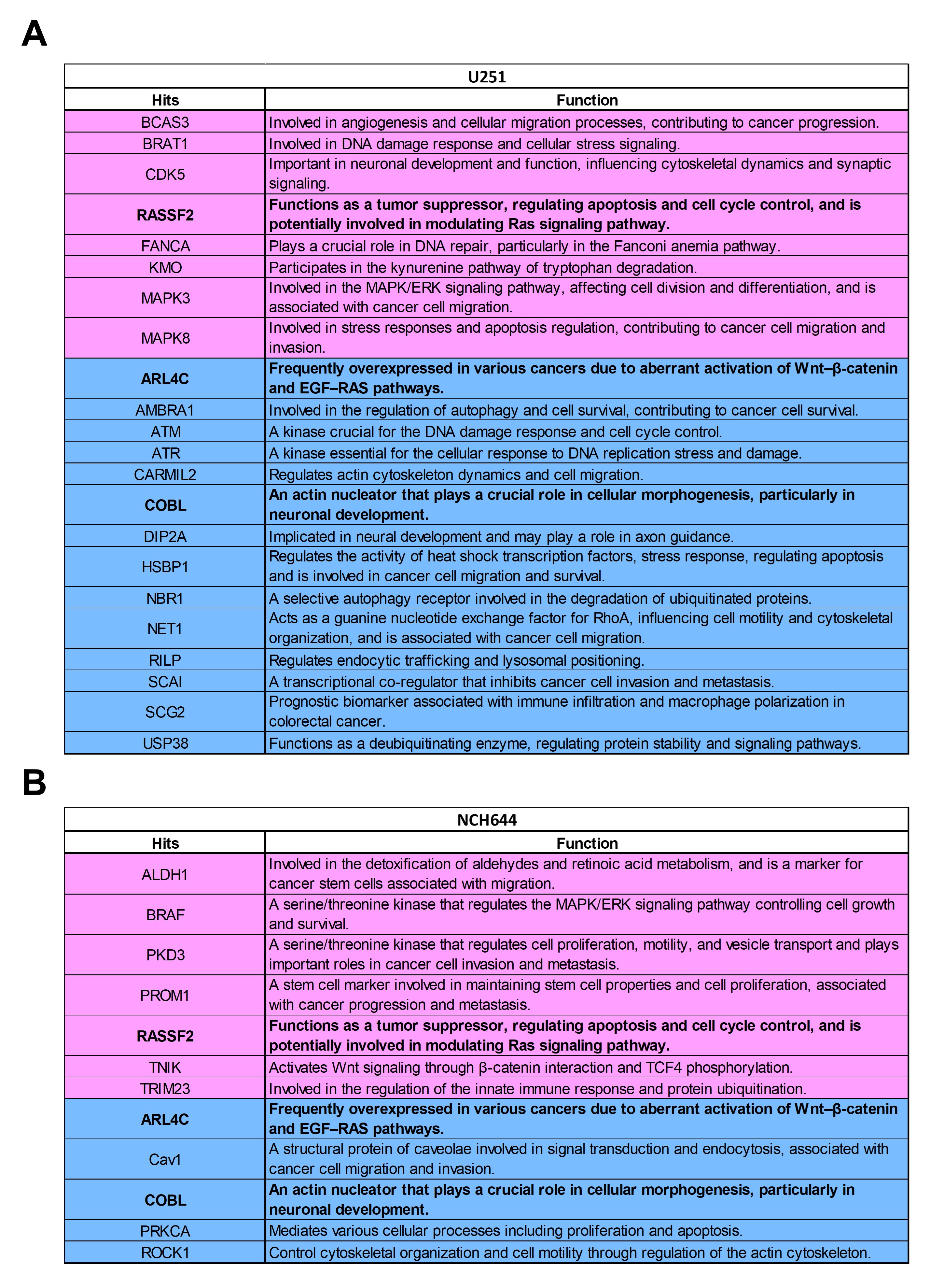

Supplement: Supplementary file 4 — Supplementary Material 4 [file 18_2024_5553_MOESM4_ESM.jpg]

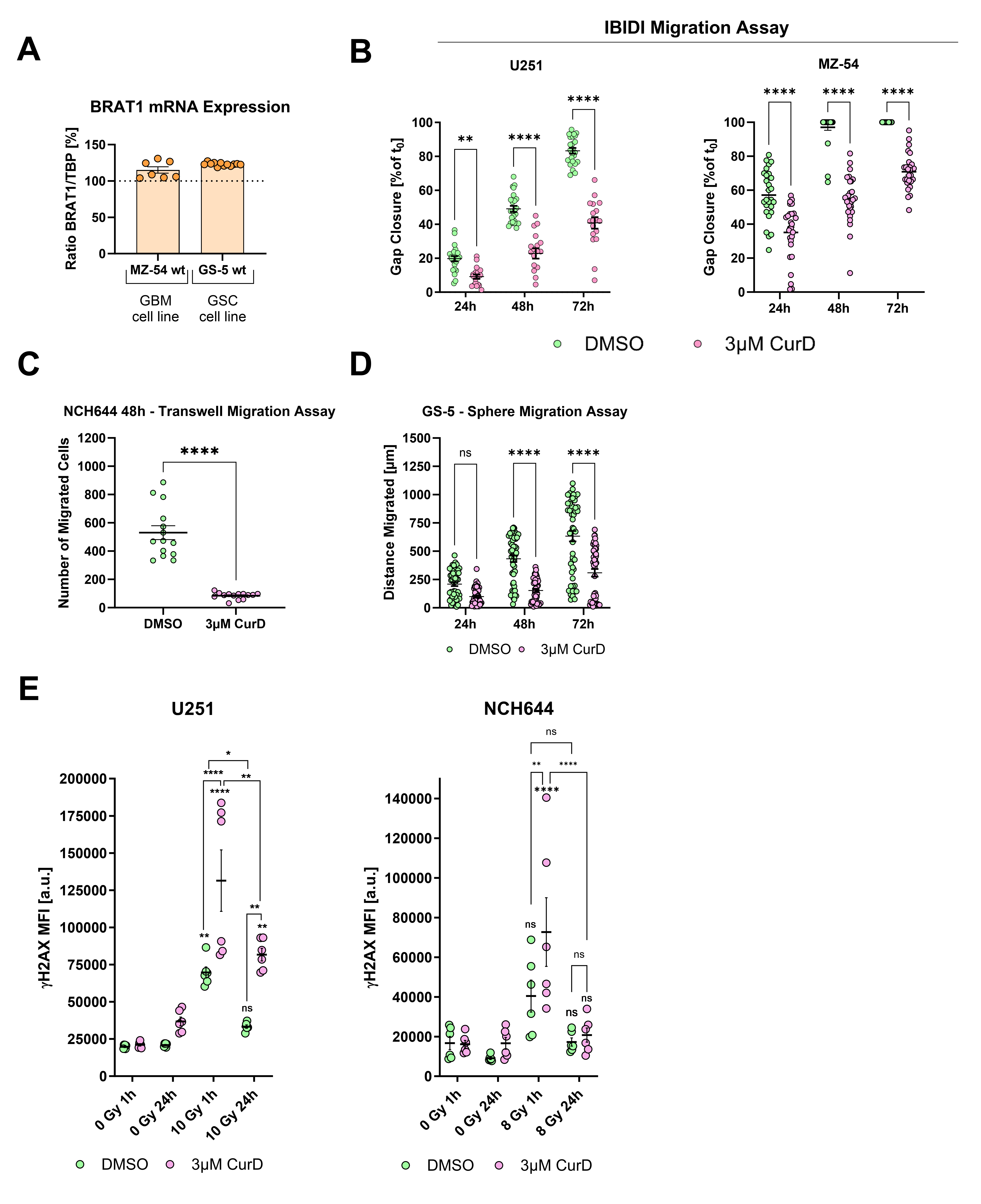

Supplement: Supplementary file 5 — Supplementary Material 5 [file 18_2024_5553_MOESM5_ESM.tif]

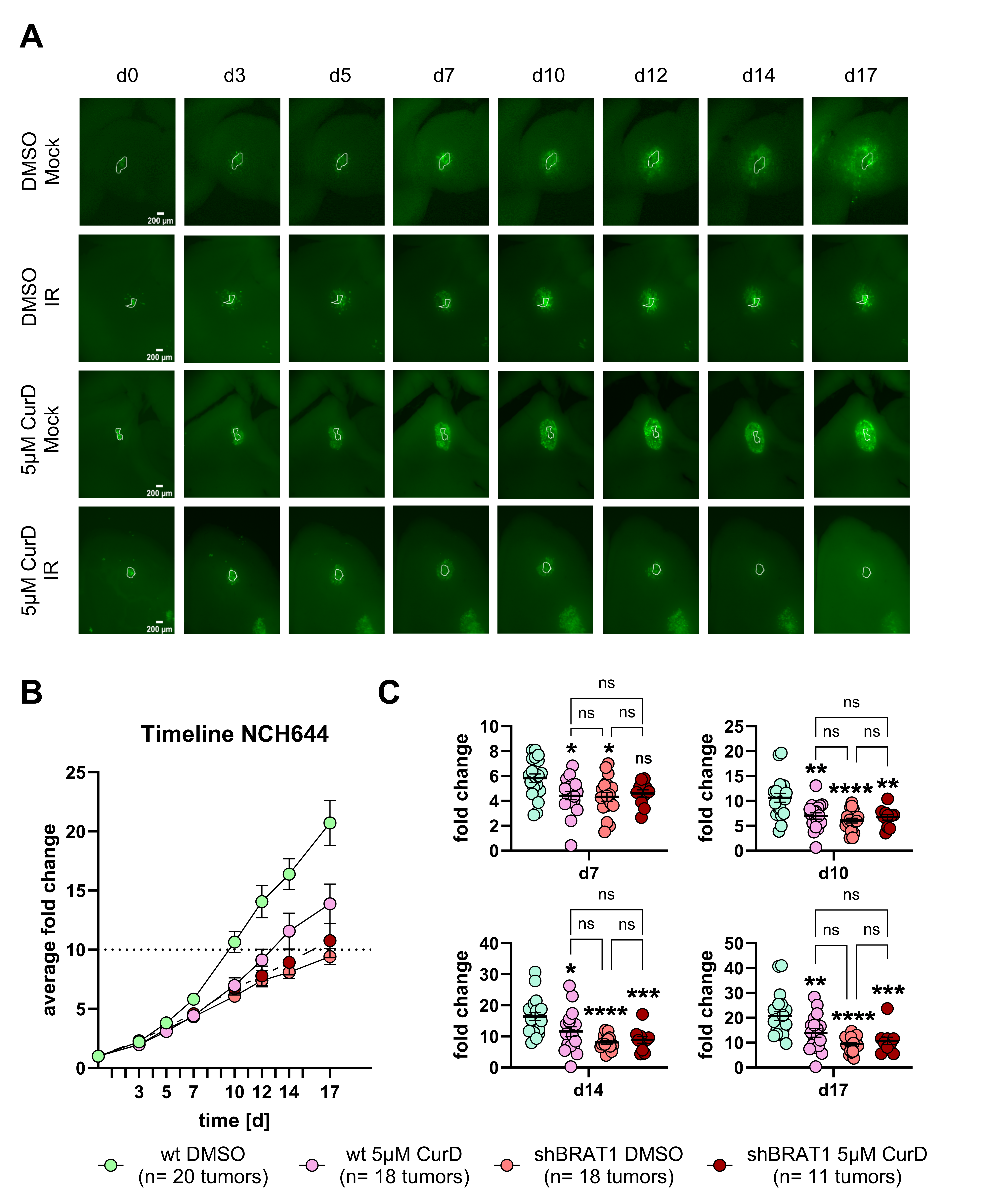

Supplement: Supplementary file 6 — Supplementary Material 6 [file 18_2024_5553_MOESM6_ESM.tif]
